# *In vivo* mouse and live cell STED microscopy of neuronal actin plasticity using far-red emitting fluorescent proteins

**DOI:** 10.1038/s41598-017-11827-4

**Published:** 2017-09-18

**Authors:** Waja Wegner, Peter Ilgen, Carola Gregor, Joris van Dort, Alexander C. Mott, Heinz Steffens, Katrin I. Willig

**Affiliations:** 1 Optical Nanoscopy in Neuroscience, Center for Nanoscale Microscopy and Molecular Physiology of the Brain, University Medical Center Göttingen, Göttingen, Germany; 2 Collaborative Research Center 889, University of Göttingen, Göttingen, Germany; 3 Max Planck Institute of Experimental Medicine, Göttingen, Germany; 4 Department of NanoBiophotonics, Max Planck Institute for Biophysical Chemistry, Göttingen, Germany

## Abstract

The study of proteins in dendritic processes within the living brain is mainly hampered by the diffraction limit of light. STED microscopy is so far the only far-field light microscopy technique to overcome the diffraction limit and resolve dendritic spine plasticity at superresolution (nanoscopy) in the living mouse. After having tested several far-red fluorescent proteins in cell culture we report here STED microscopy of the far-red fluorescent protein mNeptune2, which showed best results for our application to superresolve actin filaments at a resolution of ~80 nm, and to observe morphological changes of actin in the cortex of a living mouse. We illustrate *in vivo* far-red neuronal actin imaging in the living mouse brain with superresolution for time periods of up to one hour. Actin was visualized by fusing mNeptune2 to the actin labels Lifeact or Actin-Chromobody. We evaluated the concentration dependent influence of both actin labels on the appearance of dendritic spines; spine number was significantly reduced at high expression levels whereas spine morphology was normal at low expression.

## Introduction

Neuronal synapses are the most basic functional units of the brain. The postsynaptic part is often placed on little dendritic protrusions, the dendritic spines. Spines are highly dynamic and change their function and shape over time^1^. Spine dynamics are best observed in the living brain, where the neuronal network remains intact and native synaptic structures are preserved. Because of their minute size of 0–2 µm in length, far-field light microscopy is the only technique to visualize spines in the living tissue or organism. *In vivo* far-field light microscopy only became feasible and widely used with the advent of two-photon excitation microscopy^2^. The far-red excitation wavelength and sectioning capability rendered two-photon microscopy a superior technique to study sub-cellular structures deep in the brain tissue. However, two-photon microscopy is limited in resolution to 300 nm due to the diffraction of light and cannot resolve small details such as the thin neck of dendritic spines. In the last years, superresolution microscopy techniques have overcome the diffraction limit and are widely used to study fixed and living cells or small organisms^3^. Of all superresolution techniques, STED (STimulated Emission Depletion) microscopy is ideal for the superresolution of tissue due to its sectioning capability and the possibility to use standard fluorescent labels such as GFP or YFP, as well as its high imaging speed^4–7^. Previous work has already demonstrated that filamentous actin labelled with YFP can be resolved at a resolution < 70 nm to a depth of 40 µm in the visual cortex of a living mouse, revealing neuronal spines with unprecedented detail^8^. However, using GFP and YFP requires blue excitation (480 nm) and orange STED light (595 nm) which is not ideal for *in vivo* microscopy. Shifting the excitation and emission wavelength of the used fluorophore and therefore the laser light and fluorescence emission more to the red spectral region would bear several advantages: Far-red light is absorbed less by the tissue, causing less phototoxic stress; the lower scattering cross section improves tissue penetration^9^, and the tissue autofluorescence caused by the excitation of molecules like NADH, flavins, or haemoglobin, is reduced in red-shifted excitations when compared to shorter wavelengths^10,11^. The improvement of the penetration depth and the optimization of the light compatibility of the tissue through the use of farther red-shifted fluorescent proteins for *in vivo* STED microscopy is therefore of major interest.

We set out to study the usability of red emitting fluorescent proteins (FP) for *in vivo* STED microscopy through actin labelling, which is ubiquitously expressed in dendrites and spines. Three far-red emitting FPs have previously been utilized for STED microscopy in cultured, living cells; E2-Crimson, mGarnet, and tagRFP657. E2-Crimson is a derivative of DsRed-Express2 with an excitation and emission maximum of 611 nm and 646 nm respectively, and is a tetramer only suitable for cytosolic expression^12^. mGarnet, a derivative of mRuby (excitation: 598 nm/emission: 670 nm), is the furthest red-emitting FP used in STED microscopy so far, but exhibits a low quantum yield of only 9%^13^. TagRFP657 (611 nm/657 nm) is also monomeric with a similar quantum yield of 10% and has already been used for live cell STED microscopy^14^. When these are compared to EGFP (60%) and Citrine (76%), the quantum yields are very low for all red emitting FPs available to date.

To find a suitable protein for *in vivo* STED microscopy, the literature was screened for red-emitting proteins, specifically for their fluorescence properties. Proteins which were monomeric, with a fluorescence not below 580 nm, low photo bleaching, and a relatively high quantum yield were chosen. We built a STED microscope with a tuneable laser source and a white light excitation to adapt for the excitation and emission properties of these proteins. Based on our selection criteria and photobleaching measurements the mNeptune family was chosen^11,15^. In this study we fused mNeptune2^11^ (quantum yield 24%) to different actin binding tags: Lifeact^16^, a small peptide and Actin-Chromobody^17^, an antibody-like tag for actin. The expression of each fusion protein was recorded with STED microscopy in cultured hippocampal neurons, as well as in the cortex of a living mouse, where we were able to record time-lapse STED microscopy images over an hour at superresolution. Although mNeptune2 proved to be a good alternative for live cell STED microscopy with far-red emitting proteins, we experienced difficulties in labelling of the living mouse; proximal dendrites were much brighter than distal dendrites, rendering fluorescence imaging of layer 1 dendrites challenging. Interestingly, we also found that both actin labels, Lifeact and Actin-Chromobody, have an influence on actin morphology at high expression levels and must be deployed cautiously.

## Results

For red fluorescent protein screening and *in vivo* STED microscopy, we built an upright STED microscope similar to Willig *et al*.^8^, but with tuneable laser sources for green to red excitation and red fluorescence. The light of a Ti:Sapphire (Ti:Sa) laser, emitting pulses at 80 MHz with a pulse width of 100 fs, was split into two beams: For stimulated emission depletion, pulses were stretched to 400 ps by dispersion. A supercontinuum device was inserted into the second Ti:Sa beam to create white light^18^. Excitation light was filtered out with band pass filters and pulses were temporally aligned by an optical delay of the excitation light before the single mode fibre (cf. Methods).

We evaluated 14 different red and far-red FPs fused to Lifeact in CV-1 cells (African green monkey *Cercopithecus aethiops* kidney cells), in terms of their suitability for STED microscopy (Supplementary Table S1). Cells expressing the fusion constructs were fixed and excited at either 532 nm or 560 nm. Excitation power was adjusted according to the respective excitation spectrum to get a similar excitation probability for all FPs. The STED wavelength was 690 nm for red fluorescent proteins and 732 nm for far-red emitting fluorescent proteins, and the relative photostability after STED illumination was investigated. Of the first 9 fluorescent proteins we investigated, mStrawberry^19^, mCherry^19^, mPlum^20^, mNeptune^11^, and tagRFP657^14^ showed the lowest bleaching characteristics, indicating the greatest level of photostability (Supplementary Fig. S1a). In a separate study we included the later published mNeptune derivatives mNeptune2, mNeptune2.5, and mCardinal^11^ (Supplementary Fig. S1b). These FPs showed the furthest shift into the red emission spectrum and relatively high quantum yields out of our selection. Additionally, we tested mRuby^21^ and mRuby2^22^ (Supplementary Fig. S1c). Out of these proteins mCardinal showed the highest stability regarding photobleaching, followed by mNeptune and mNeptune2 which were comparable (Supplementary Fig. S1b). Due to the higher quantum yield of mNeptune2 (24%) in contrast to mCardinal (19%) and mNeptune (23%), we decided to use mNeptune2 as a far-red fluorescent protein in the following STED experiments.

### Live cell STED microscopy of cultured neurons

**Figure 1:**
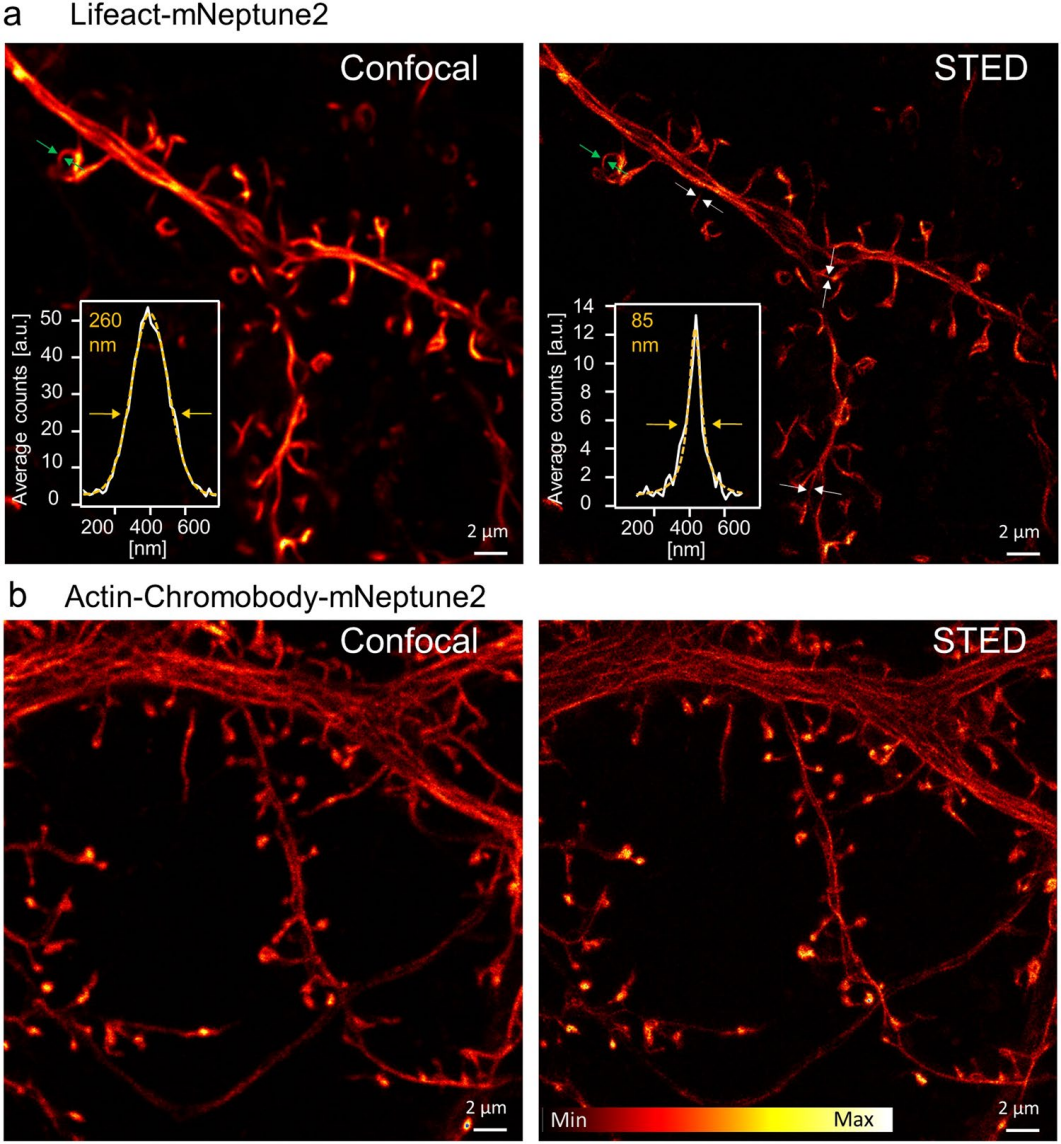
Live cell STED microscopy of neurons expressing F-actin labels fused to mNeptune2. Neurons were transduced with (**a**) Lifeact-mNeptune2, and (**b**) Actin-Chromobody-mNeptune2, at 11 days *in vitro*. Living neurons were measured with an excitation wavelength of 586 nm shown as the conventional confocal image (left) and with an additional STED laser at 775 nm resulting in the superresolution STED image (right). Inset of (**a**): Line profiles, taken across the indicated filament (green arrows); average of three lines of raw data with Lorentz fit (STED) or Gaussian fit (Confocal) and full-width at half-maximum (FWHM). In STED-mode the FWHM measured at 4 positions (85 nm green arrow, 86 nm, 87 nm, and 74 nm, white arrows) averages to 83 nm.

To confirm the suitability of mNeptune2 for live cell STED microscopy in neurons, we prepared adeno-associated viral particles (AAV) of mixed serotype 1 and 2. The particles carried either the coding sequence of Lifeact-mNeptune2 or Actin-Chromobody-mNeptune2, under the control of the human Synapsin 1 promoter (hSyn)^23^. These AAVs were then added to cultured neurons and incubated. Eleven days post-transduction, we investigated F-actin labelled with mNeptune2 in the living neuron (Fig. 1). STED microscopy on the samples revealed actin filaments with an average of 83 nm full-width at half-maximum (FWHM) (Fig. 1). Without knowing the exact size of the structure, we estimate the resolution to be an upper estimate of ~80 nm assuming at least one single F-actin filament of 5–9 nm in size^24^. We neither observed cytotoxic effects caused by the actin-labels fused to mNeptune2 nor phototoxic effects by the illumination light; cells were alive and repeated illumination did not change the morphology. The photostability, maximum available brightness, and absence of phototoxicity renders mNeptune2 suitable for long term time-lapse *in vivo* STED microscopy.

### *In vivo* time-lapse STED microscopy

**Figure 2:**
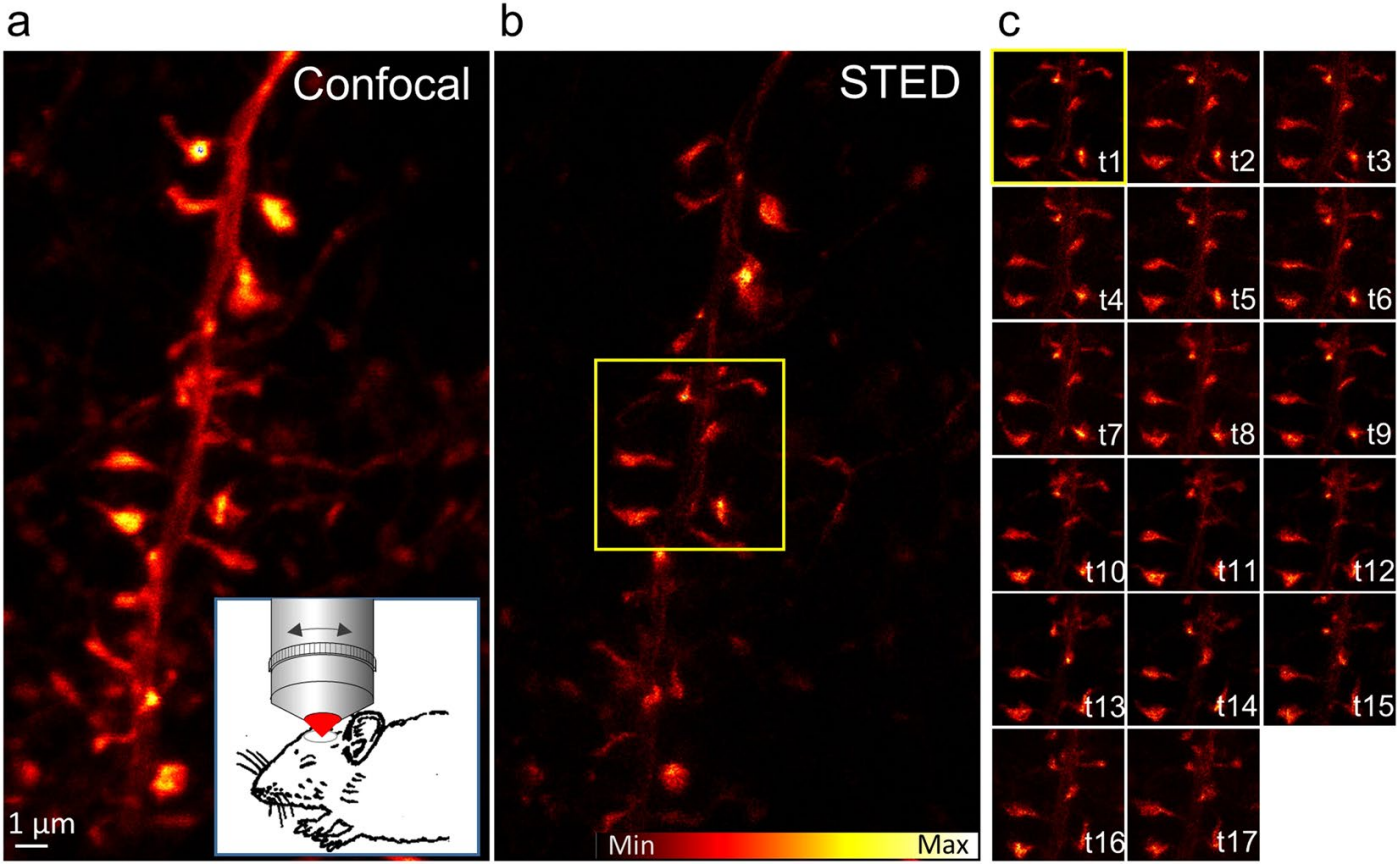
*In vivo* STED microscopy of filamentous actin marked with Lifeact fused to the far-red emitting fluorescent protein mNeptune2. A dendritic branch at 6 µm depth of the visual cortex is recorded at 560 nm excitation in the confocal mode (**a**) and by an additional laser at 732 nm for stimulated emission depletion (STED) in superresolution mode (**b**). The correction collar of the objective was used to optimize the resolution in the tissue^8^. (**c**) Image section of the marked area in (**b**) of 17 time points (t) recorded within 55 min, each with a maximum intensity projection of 4 z-slices of raw data 500 nm apart. For the whole segment see the Supplementary Movie S1.

After the successful imaging of live neurons with mNeptune2, the applicability of these constructs in living mice was subsequently investigated. We injected ~150 nl of concentrated AAV into layer 5 of the visual cortex of C57BL/6 wt mice. After ~3 weeks a craniotomy was performed by drilling a 2 mm Ø large opening into the bone above the visual cortex of an anesthetised mouse and closed the opening with a cover glass^8^. The mouse was then transferred to the upright STED microscope for *in vivo* imaging. A view through the transcranial window revealed dense labelling of dendritic protrusions. Used constructs are under the control of the hSyn promoter, which is described to be neuron-specific^23^, and labelled non neuronal cells were never detected. Figure 2 shows the confocal and STED image of a dendrite with spines located in layer 1 of the visual cortex. The STED superresolution image of a dendrite reveals more detail, in the delicate morphology and distribution of the actin filaments in the dendritic branch (Fig. 2b), than is visible in the corresponding confocal image (Fig. 2a). To estimate the resolution we measured the FWHM of line profiles (average of 5 lines) at 7 different positions in the image. With an average of 79 nm the FWHM of small structures was very similar to the live cell STED recordings (Fig. 1) also indicating a resolution below 80 nm. Importantly, with time-lapse STED microscopy we were able to record the plasticity and the changes of spine morphology, over a time course of 1 h in the living mouse brain on the nanoscale, without any visible signs of phototoxicity during that time period (Fig. 2c and Supplementary Movie S1).

### Differences in Actin-mNeptune2 labelling of proximal and distal dendrites

**Figure 3:**
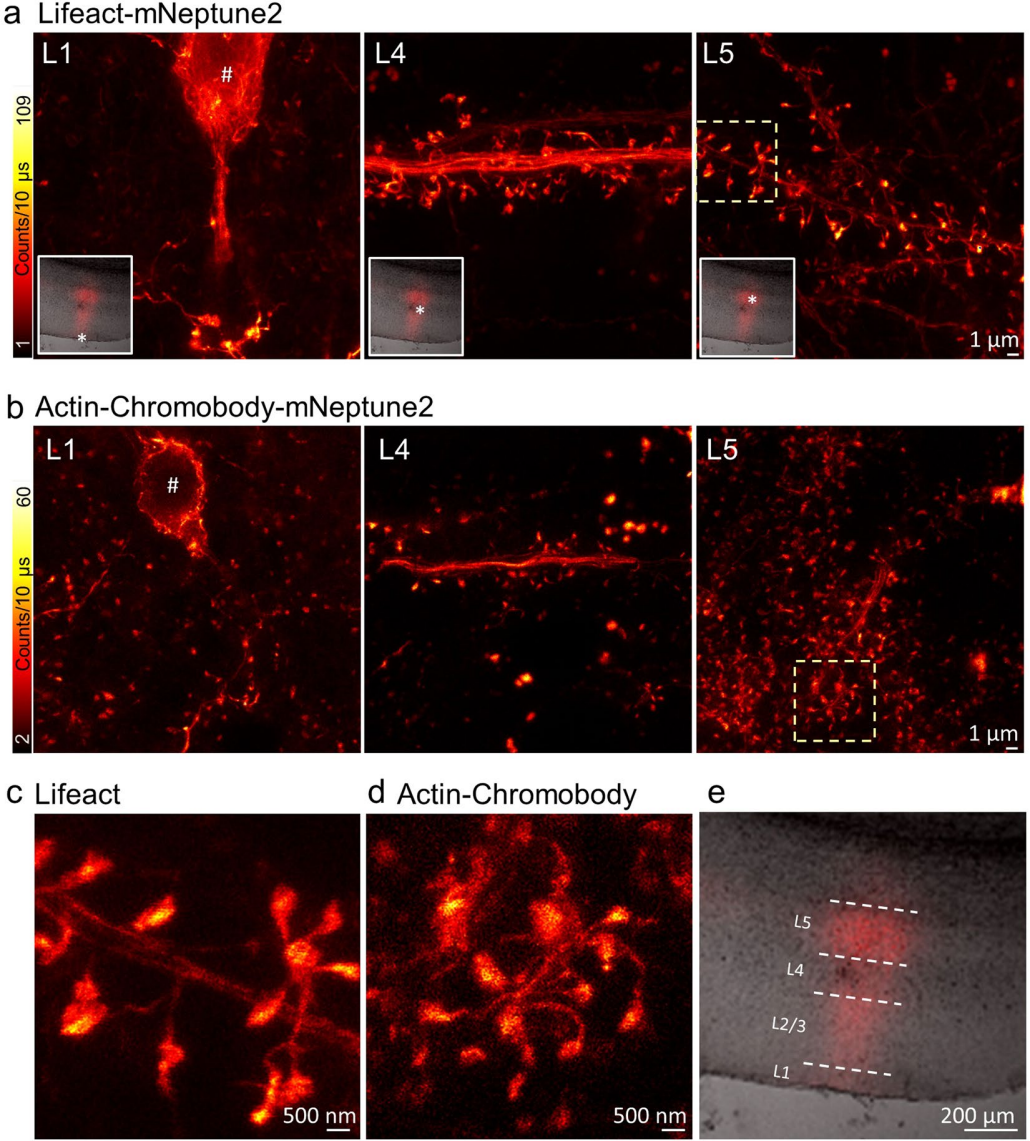
STED microscopy in different cortical layers using Lifeact and Actin-Chromobody to highlight filamentous actin. (**a**) Lifeact-mNeptune2 expressed in a superficial neuron in layer 1 (L1) shows a bright actin label while the surrounding spines are rather dark (# indicates a cell body). Apical dendrite of layer 5 which passes layer 4 (L4) is rich in actin bundles in the dendritic shaft and spines. Layer 5 (L5) exhibits dendrites with densely packed dendritic spines. All images were recorded with the same settings and scaled to the same maximum and minimum photon counts for comparison of the fluorescence expression levels. White boxes show a widefield overview image with the area of measurement indicated by a star. (**b**) Actin-Chromobody fused to mNeptune2 is highly expressed in rare neurons of L1 and dark in surrounding spines (left). STED microscopy reveals bright apical dendrites in L4 (middle) and densely packed dendritic spines in L5 (right). (**c,d**) Magnified view of the yellow boxed area (**a**,**b** respectively), showing the superresolved spine morphology in greater detail. (**e**) Magnification of the overview images in (**a**), showing an overlap of the bright-field (grey) and fluorescence wide-field (red) microscopy image.

Our *in vivo* STED and confocal recordings of layer 1 (L1) of the visual cortex showed inhomogeneous brightness of the labelling. Some dendritic branches were bright, whereas neighbouring parts were very dark and thus not eligible for STED (Fig. 2). To understand this finding, we perfused the mouse transcardially after the *in vivo* experiment with 4% paraformaldehyde (PFA) in PBS and subsequently dissected the brain. Several 70 µm thick sections around the initial injection site of the AAV were prepared using a Vibratome (VT1000S, Leica Microsystems, Wetzlar, Germany) and studied microscopically. Overview widefield fluorescence images showed a dense fluorescence label of Lifeact-mNeptune2 in layer 5 (L5) and up to L1 (Fig. 3a insets, Fig. 3e). We recorded STED microscopy images at cortical layer L1, L4, and L5, indicated by a star in the insets of Fig. 3a, revealing the dendritic actin distribution at superresolution. In L5, the dendritic branches exhibited a bright fluorescence with densely packed spines. L4 contains only inhibitory neurons which do not express our construct. Therefore, we can easily identify L4 as the layer without any labelled neuronal soma containing only thick apical dendrites of L5 which pass through this layer. L1 (Fig. 3a) showed a similar labelling pattern as previously observed *in vivo*; a brightly fluorescent neuron (identified by its bright soma marked with #) with some bright protrusions is surrounded by several much darker dendrites with numerous spines which could almost be confused as background. A comparison of the brightness of dendritic protrusions at different layers shows that those of the marked surface neuron in L1 (Fig. 3a) are comparable with the brightness of the dendritic protrusions in L5 and L4 (Fig. 3a). While all bright dendritic protrusions of L1 could be attributed to a L1 soma, the dark dendritic protrusions surrounding the soma in L1 of Fig. 3a are most likely distal branches of L5 pyramidal neurons, which are much dimmer than the proximal dendritic protrusions of L5 (Fig. 3a L5). Often, our injection method of AAV into L5 labelled pyramidal neurons in layer 2/3 as well. However, this did not change our observation of bright and dark dendritic structures in L1. Due to this observation, and the fact that it is difficult to distinguish L1 and L2/3, we did not specify the intensity of L2/3 labelling in this study. To exclude the possibility that the imbalance of labelling brightness was due to the actin label with Lifeact, we repeated this experiment with the Actin-Chromobody. Figure 3b shows a very dense labelling of spines with Actin-Chromobody-mNeptune2 in L5. The cortical slice (Fig. 3b) as well as the *in vivo* STED experiments (Supplementary Fig. S2a) revealed brightly fluorescent dendritic branches and spines in L1, but also many spines of much weaker fluorescence. Again, the brightly fluorescent spines can often be attributed to an L1 soma, whereas the weak fluorescence is much more likely to be attributed to distal dendrites of L5 neurons and is considerably weaker than the labelling of L5 proximal dendrites (Fig. 3b L5). However, a magnification of the STED microscopy images, with Lifeact (Fig. 3c) and Actin-Chromobody (Fig. 3d) labelling in L5, shows a bright labelling of filamentous actin in the dendritic shaft and spine necks, as well as a dense accumulation of actin in the spine head. To assess if the decrease of labelling in distal dendrites occurs due to the properties of the FP, we repeated this experiment with the expression of Lifeact-GFP (Suplementary Fig. S3). When compared to Lifeact-mNeptune2, the expression of Lifeact-GFP shows L1 dendritic protrusions of similar brightness as in L5 for the GFP fusion protein, whereas L1 of the mNeptune2 expressing mouse shows a large number of dark dendrites which could indicate a limited transport of Lifeact-mNeptune2 and Actin-Chromobody-mNeptune2 to distal dendrites (Supplementary Fig. S3).

### (Over) expression of Lifeact or Actin-Chromobody alters spine morphology

**Figure 4:**
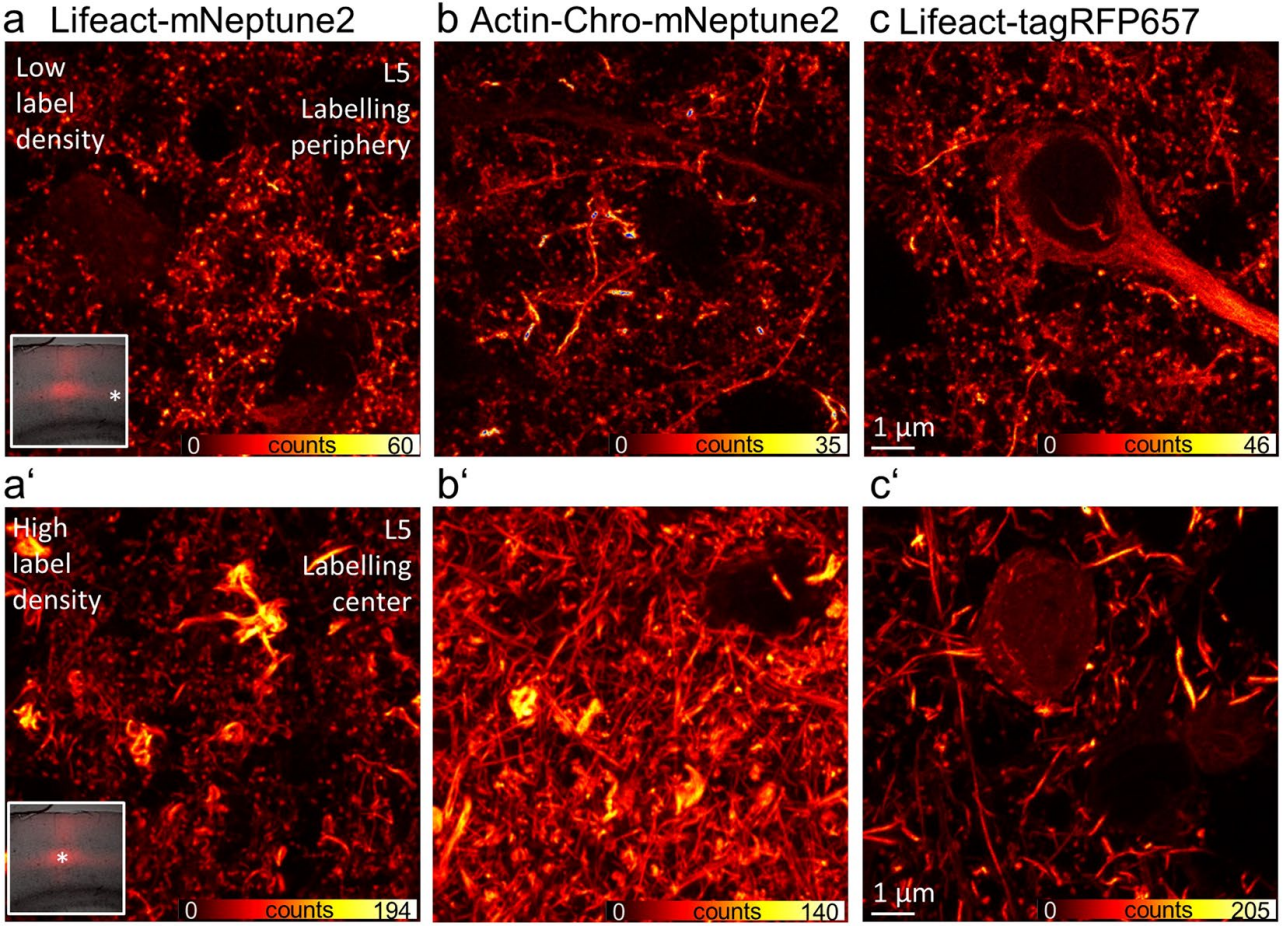
Dendritic morphology at high and low expression levels of actin binding fusion proteins. Confocal scans of a brain slice at the region indicated by a star in the boxed overview image (**a**,**a’**). (**a**) Lifeact-mNeptune2 expression in L5 in the periphery of the fluorescent area shows plenty of dendritic spines which are abolished in the central region (**a’**). (**a’**) Bright accumulation of fluorescent fusion proteins which are not present at lower expression levels in the periphery of the labelling (**a**). (**b**) Actin-Chromobody-mNeptune2 expression in L5 highlights spines and dendrites; rare overexpressing dendrites. (**b’**) No spines are visible at high expression. (**c**) Actin labelling of spines and a cell body at low expression compared to thick bundles of actin in dendritic shafts without spines at high expression levels of Lifeact-tagRFP657 (**c’**) within the same brain slice. Note the increase in brightness between the centre and the periphery of the labelling.

The brightly fluorescent proximal dendrites close to the soma of L5 pyramidal neurons are at a depth of ~500 µm in the living animal, which is not accessible with *in vivo* STED microscopy. Therefore we tackled improving the brightness of apical dendrites by raising the expression level of the fusion construct through increasing the AAV virus titre. Unfortunately, an increased expression of Lifeact and Actin-Chromobody resulted in a complete loss of spines and partial accumulation of FPs. To evaluate these influences on spine morphology, we expressed different fusion constructs of actin labels in L5 of the visual cortex via AAV transduction. Concentrated AAVs of the hSyn-Lifeact-mNeptune2 construct were injected into the mouse brain. After ~3 weeks of expression, we perfused the mouse transcardially with 4% PFA in PBS and prepared brain slices of the transfected regions to analyse them with fluorescence microscopy. Figure 4a shows a confocal image of a typical labelling at the periphery of the transfected region. This region of low FP expression is densely packed with multiple dendrites rich in spines. In contrast, the central region of high FP expression (note the increase in fluorescence signal) shows a dense labelling and the accumulation of protein at some spots, but very few spines (Fig. 4a’). To test if these expression artefacts are due to the Lifeact binding to actin, we expressed hSyn-Actin-Chromobody-mNeptune2, which contains a completely different type of actin label. Comparable to the expression of the Lifeact construct, the expression of the Actin-Chromobody also leads to thick bundles of actin in the central region and no or low numbers of dendritic spines (Fig. 4b’). At the periphery of the labelled region, and therefore at lower expression levels, dendritic spines were conserved and only in some dendrites accumulation of FPs was visible. Additionally, we tested if these artefacts are caused by the fluorescent protein mNeptune2 itself. In order to examine this, Lifeact was fused to tagRFP657^14^, a FP which is emitting fluorescence in a similar wavelength range as mNeptune2, allowing for the same microscope settings to be used, and expressed it *in vivo* (Supplementary Fig. S2b). Again, at low expression levels spines appeared normal (Fig. 4c), but their abundance was drastically reduced at higher expression levels in the central region (Fig. 4c’). We repeated this experiment with the same constructs in neuronal cultures, expressing different levels of Lifeact and Actin-Chromobody fused to mNeptune2 or tagRFP657, and saw the same effect: Spines disappearing at high expression levels (Supplementary Fig. S4a-c). We also fused the actin binders to the better characterized GFP and YFP (Supplementary Fig. S4d,e). With these green and yellow fluorescent proteins we again observed no spines at high expression levels, indicating that this is not a unique characteristic of red or green emitting proteins but rather the actin binding itself or FP expression in general. Additionally, we increased the expression of the actin binding fusion protein of mNeptune2 and tagRFP657 by using the higher expressing hybrid form of the CBA promoter (CBh)^25^ which also resulted in a loss of spines (Supplementary Fig. S4f,g). To ensure that these findings were not the result of differences in the quality of the neuronal cell culture, we fixed and labelled cultures of hippocampal neurons with phalloidin, the “gold standard” of F-actin labelling, showing neurons rich in dendritic spines (Supplementary Fig. S5). To study the influence of the FP alone on the loss of spines, we transduced neurons with different constructs only expressing either GFP, tagRFP657, or mNeptune2.5 (without any actin binders). After 8–11 days the neurons were co-labelled with phalloidin (Atto 633 or Alexa Fluor 488). Two-colour confocal imaging detected cytosolic and nuclear localization of the FPs as well as normal spine morphology at low to high expression levels of the FPs (Supplementary Fig. S6). To conclude, low level expression of Lifeact-FP or Actin-Chromobody-FP visualizes normal morphology of neuronal F-actin, and the observed expression artefacts of these actin labels do not appear with pure FP expression.

## Discussion

STED microscopy is ideal for studying synaptic processes in the living organism due to its superresolution capability and the relative high imaging speed. It could be best achieved by using fluorescent proteins which have an excitation and emission peak in the red or far-red wavelength. This reduces background signals from the autofluorescence of endogenous fluorophores, causes less phototoxic stress, and offers a lower scattering cross section for improved tissue penetration. Out of 14 tested proteins, we have applied the far-red proteins mNeptune2 and tagRFP657 in live cell and *in vivo* STED microscopy experiments. No signs of phototoxicity were observed, at either 560 or 586 nm excitation, nor with the 732 or 775 nm STED light, which renders these ranges of wavelengths ideal for *in vivo* STED microscopy. Moreover, the photostability of mNeptune2 was sufficient to study actin dynamics in a neuron, within the visual cortex of L1 utilising time-lapse *in vivo* STED microscopy over a time scale of ~1 h. To visualize actin, we used AAVs with two different kinds of actin labels: Lifeact and Actin-Chromobody fused to the far-red fluorescent proteins. High expression levels of both actin labels resulted in artificial actin distribution, and finally in the total loss of spines. This effect was highly concentration dependent, and was not observed at low expression levels. The change in spine morphology was not caused by the FP itself or potential impurities of the AAVs, because AAV mediated FP expression alone did not alter spine abundance or morphology even at high expression levels. Whether expression of Lifeact or Chromobody alone causes the observed artefacts needs to be investigated in the future, but will be challenging because Lifeact, for example, is only 17 aa short, and might be degraded quickly by the cellular degradation machinery. Although Lifeact is widely used, not many publications report Lifeact expression artefacts. Recently it was shown that Lifeact affects the actin assembly in yeast cells when fused to EGFP or mCherry and possible mechanisms of actin assembly disturbance by Lifeact were investigated^26^. Furthermore, it has been shown that Lifeact interferes with actin dynamics in the plant *Arabidopsis thaliana* as well as in filamentous fungi^27,28^. So far no Lifeact overexpression artefacts have been shown in the living mouse. Other more frequently used actin labels, e.g. F-tractin, the minimal F-actin binding domain (amino acids 9–40) of rat inositol trisphosphate 3-kinase A (ITPKA), have also been shown to induce abnormal spine elongation at high level expression^29,30^. Moreover high expression of the F-actin binding protein Utrophin (calponin homology domains of human ubiquitous dystrophin), has also been shown to cause severe actin defects^31^, indicating that the expression of actin labels needs to be precisely controlled in any given cellular system. An overview of pros and cons of different actin visualisation tools has been briefly summarized in Melak *et al*.^24^.

For both of our constructs, Lifeact as well as Actin-Chromobody, we determined the optimal amount of AVV resulting in low expression levels that resembled native spine morphology as indicated by non-transduced wildtype neurons labelled with fluorescent phalloidin. Interestingly, we observed that the actin structures labelled with Actin-Chromobody and Lifeact might not be identical, as Lifeact more frequently highlights actin in the dendritic shaft. This may occur due to alterations in their affinity to different kinds of actin bundles. This has already been identified for the F-actin binding protein ITPKA, where amino acids 1–66 bind F-actin mainly in spines, and amino acids 9–52 are predominantly enriched in dendrites^29^.

Based on our experiments, we showed that mNeptune2 is suitable for live cell STED imaging. Our study also indicates however, that actin, highlighted with fusion proteins of far-red FPs, is much darker in distal dendrites compared to proximal dendrites. Exchange of the far-red FP with GFP resulted in greater homogeneity of labelling, indicating that this effect is related to the properties of the red FP. We could not find any published data of such an observation and cannot explain it so far, however, all far-red emitting FPs are related and as such may demonstrate the same inhomogeneous labelling. We expressed the red fusion proteins for 3–4 weeks in the living mouse and did not see any signs of toxic effects, neither in the behaviour of the mice, nor in the morphology of dendrites and spines. A remaining drawback of far-red FPs is the lower brightness compared to GFP variants. A future option for *in vivo* STED microscopy might well be novel red emitting organic dyes which are coupled via SNAP-tag or Halotag. Therefore, an ideal red emitting organic dye should be cell permeable, nontoxic, and have the ability to pass the blood brain barrier. The now commercially available fluorogenic F-actin label SiR-Actin (Spirochrome AG, Stein am Rhein, Switzerland), has been successfully used in live cell STED imaging^32,33^. This is not, however, an acceptable alternative for STED microscopy in the living mouse, as all actin will be marked, leading to a dense labelling even in non-neuronal cells. As such, a genetically encoded actin marker, whose expression pattern can be controlled, e.g. via a promoter or Cre recombinase^34^ induced expression, which could be coupled to a bright far-red STED suitable fluorescent protein or dye, would be of outstanding interest for *in vivo* STED microscopy.

## Methods

### Plasmid construction

Fusion proteins of Lifeact (LA) MGVADLIKKFESISKEE with the far-red fluorescent proteins mNeptune2 and tagRFP657 were generated by using PCR. cDNA of mNeptune2 and tagRFP657 were amplified with a forward primer 5-TAGTACACCGGTCGCCACC**ATG**NNNNNNNNNNNNNNN-3′ (N = nucleotide of corresponding cDNA sequence) including an AgeI restriction site and a reverse primer 5′-CATGAATTC **TTA**NNNNNNNNNNNNNNN-3′ including an EcoRI restriction site. After purification, PCR fragments were digested and ligated into the equally digested pAAV-hSyn-LA-EYFP plasmid^8^, to create the constructs pAAV-hSyn-LA-mNeptune2, and pAAV-hSyn-LA-tagRFP657 (linker sequence between LA and fluorescent protein: GDAPVAT). Plasmids including the actin camelid antibody were cloned as follows: cDNA coding for the camelid antibody against actin was PCR amplified from the Actin-Chromobody® plasmid (TagGFP35, Chromobody, Chromotek, Planegg, Germany) by using forward primer 5′-GATCGCATGCCTTAAG **ATG**GCTCAGGTGCAGCTG-3′, which adds restriction sites for SphI and AflII, and reverse primer 5′-CTAGGGTACCACCGGTGGCACCACTACCTCTTGAGGAGACGGTGAC-3′, which adds restriction sites for KpnI and AgeI as well as a 4 amino acid linker (Gly-Ser-Gly-Ala) to the 3′-end of the camelid antibody coding sequence. The amplified sequence was purified, digested with SphI and KpnI and sub-cloned into the vector pQE30 (Qiagen, Hilden, Germany) that was opened accordingly. mNeptune2 coding sequence was PCR amplified with forward primer 5′-CTAGCTGCAGCCACTAGTGGTAGTGGTGCCGTGTCTAAGGGCGAAGAGCTG-3′, omitting the start codon of the FP and adding restrictions sites for PstI/SpeI as well as a 4 amino acid linker (Gly-Ser-Gly-Ala) to the 5′-end, and reverse primer 5′-TCGAAAGCTTTTACTTGTACAGCTCGTCC-3′, which adds a HindIII restriction site to the 3′-end of the fluorescent protein. The PCR fragment was digested with PstI and HindIII and ligated into the equally opened pQE30 vector already harbouring the cDNA of the camelid antibody, resulting in the vector pQE30-Chromobody-mNeptune2. Together with the remaining native multiple cloning site sequence of the pQE30 plasmid the additional sequences at the 3′-end of the camelid antibody and the 5′-end of the fluorescent protein form a 20 amino acid linker between the antibody and the fluorescent protein (FP). Subsequently, the complete Chromobody-mNeptune2 construct was amplified by PCR with the forward primer 5′-GTACGGATCCATGGCTCAGGTGCAGCTGGTGGAG-3′ and the reverse primer 5′-CGATGAATTCTTACTTGTACAGCTCGTCCATGCC-3′, flanking the construct with a BamHI and an EcoRI restriction site. The PCR product was digested with both restriction enzymes, and ligated into plasmid pAAV-hSyn-LA-EYFP, which was digested accordingly, resulting in pAAV-hSyn-Chromobody-mNeptune2.

### AAV production

AAVs were produced in HEK293-FT cells (human embryonic kidney cell line, Gibco, ThermoFisher scientific, cat. No. R700-07, Darmstadt, Germany) by using *Trans*IT®-293 Transfection Reagent (Mirus Bio LLC, Madison, WI). In brief, cells were seeded after manufacturer’s recommendations and transfected with the following four plasmids (with a molar ratio of 2:2:1:1) to get virus particles of mixed serotype 1/2 (AAV1/2): pAAV-hSyn-LA-FP, pFdelta6 an adenovirus helper plasmid, pH21 enclosing the AAV1 rep and cap sequences, and pRV1 containing replication and capsid proteins of serotype 2. Two days after transfection, cells were resuspended in lysis buffer (150 mM NaCl, 50 mM Tris-HCl, pH 8.5) and disrupted by 3 freeze-thaw cycles. After DNaseI (ThermoFisher Scientific) treatment for 30 min at 37 °C, cellular debris were removed by centrifugation at 1200 g for 10 min, followed by 5 min at 3300 g. Supernatant was again centrifuged for 2 h at 46,000 g. The virus particle containing pellet was air dried and resuspended in sterile filtered PBS (pH 7.4).

### Mouse surgical procedure and virus transduction

All mouse experiments were performed according to the guidelines of the national law regarding animal protection procedures and by the responsible authorities, the Niedersächsisches Landesamt für Verbraucherschutz.

AAV transduction was performed by stereotactic injection as previously described^8^. Mice were anesthetized by *i.p*. injection of 60–80 mg pentobarbital sodium (in 0.9% NaCl) per kg of body weight. Approximately 150 nl of AAV were injected with a micropipette connected to a picospritzer (Tooheyspritzer; Toohey Company, Fairfield, NJ) which was inserted into the skull at a position 200 µm frontal of the lambdoid suture and 2.5 mm left to the sagittal suture. The micropipette was fed 700 µm into the brain under an angle of 30° from the horizontal plane. The skin was then closed with 2–3 stitches and the mice were kept on a heating plate until waking. After full recovery the mice were kept at the animal care unit for 3–4 weeks until the final STED experiment was performed.

The surgical procedure for the *in vivo* experiment follows the previously described protocol^8^. In brief, anaesthesia was initiated by pentobarbital to cannulate the left jugular vein. Anaesthesia was continued with 75 mg·kg^−1^·h^−1^ methohexital sodium (Brevimytal®, HIKMA) *i.v*. throughout the duration of the experiment. The mouse was artificially ventilated through a T-shaped tube in the trachea and paralyzed with pancuronium bromide. Vital functions and depth of anaesthesia were controlled throughout the experiment by recording the ECG, measuring O_2_ saturation of the blood and body temperature. A circular opening (2 mm in diameter) was drilled into the skull with the centre above the former virus injection site. The bony plate was removed together with the attached dura mater. The arachnoid membrane was then removed with a fine forceps. At the edge of the hole we placed a small tube to be able to extract excess cerebrospinal fluid. The opening was closed with a coverslip of 6 mm diameter glued to the skull with tissue glue (Histoacryl®, B.Braun, Melsungen, Germany).

### Primary hippocampal neuronal cell culture, transduction, and imaging

Primary neuronal cell cultures were prepared from rat and mouse hippocampus. Cultures of rat hippocampal neurons were generated from P0-P1 Wistar rats of mixed sex and were prepared as previously described^36^. Hippocampal mouse neurons were maintained from P0 C57Bl6/N mice of mixed sex and prepared as described in Burgalossi *et al*.^37^ under the section “Preparation of hippocampal neuronal autaptic cultures”. Primary hippocampal neurons were plated on cleaned, Poly-L-lysine (Sigma-Aldrich cat. No. P4707, Darmstadt, Germany) coated coverslips, without astrocytes. Cultures were incubated at 37 °C in a humidified atmosphere with 5% CO_2_. Neurons were transduced at 11 days *in vitro* (DIV) with AAV1/2-hSyn-LA-mNeptune2 and AAV1/2-hSyn-Actin-Chromobody-mNeptune2 and live imaged at an age of 22 DIV. Live cell imaging was performed at room temperature in normal neuronal culture media.

### *In vivo* STED microscope

We built a STED microscope with a tuneable excitation and STED light source to optimally accomplish stimulated emission of the red-emitting fluorescent proteins that were under analysis. Both the excitation and STED beam was provided by a single Ti:Sa laser (MaiTai; Spectra-Physics, Darmstadt, Germany). After passing an optical isolator (Model 713, conoptics, Danbury, CT) the beam was split into two beams: For stimulated emission, the ~100 fs long MaiTai pulses were stretched by dispersion in a 40 cm long glass rod and focused into a 120 m polarization-preserving fibre (OZ Optics, Ottawa, Canada). With the second beam, white light was generated by passing the light through a supercontinuum device (FemtoWHITE800, NKT photonics, Birkerød, Denmark). Spectrally filtered with a ET560/20 M (Chroma, Bellows Falls, VT) and additional HQ550/40 M (Chroma) bandpass, the excitation beam was spatially filtered and temporally stretched by passing along a 15 m long polarization-preserving fibre (OZ Optics). Excitation light was separated from fluorescence by a custom-made long-pass dichroic mirror (580DCXRU, Chroma). After passing through a vortex phase plate (RPC Photonics, Rochester, NY), the STED beam was co-aligned with the excitation beam using a short-pass dichroic (z720SPRDC, Chroma). Passing a Yanus scan head (Till Photonics-FEI, Gräfelfing, Germany) for (x,y)-beam scanning and a quarter wave plate, the excitation and STED beam entered an upright microscope stand (DM6000, Leica Microsystems, Wetzlar, Germany) and were focused by a 1.3 NA objective lens (PL APO, 63x, glycerol; Leica Microsystems) into the mouse cortex. Z-scanning was accomplished by moving the objective with a piezo (MIPOS 100PL, piezosystem jena, Jena, Germany). De-scanned fluorescence was filtered with an ET645/75 M (Chroma) band-pass and focused on a multimode fibre of 62.5 µm diameter for confocal detection connected to an avalanche photodiode (APD, PerkinElmer, Waltham, MA). Images were acquired with Imspector software (Abberior Instruments, Göttingen, Germany).

### Live cell STED microscope

Cultured neurons were imaged on a home-built STED microscope featuring an inverted microscope stand to facilitate the handling of the cells attached to a cover glass. Inspired by the publication of Göttfert *et al*.^38^, we utilized excitation light at 586 nm which was branched off from the white light of the *in vivo* STED and filtered out with a band pass filter (586/20, Semrock, Rochester, NY), before being focused into a 10 m long optical fibre. The 775 nm STED laser (Katana 08 HP, OneFive GmbH, Regensdorf, Swiss) was focused into a 10 m long optical fibre (OZ Optics) and the output was shaped by a spatial light modulator(Abberior Instruments) before being combined with the excitation beam by a short-pass dichroic (T750spxrxt, Chroma). To overlap the pulses in time, the STED laser is triggered by the Ti:Sa laser via a TTL-signal. Both beams were (x,y)-scanned by two galvanic mirrors (MicroMax 673XX, Cambride Technology, Bedford, MA) and pass a quarter wave plate before entering an inverted microscope stand (DMi8, Leica Microsystems) and then being focused by a 1.4 NA objective (HCX PL APO 100x/1.40 OIL STED, Leica Microsystems), which was based on a z-scanning piezo (MIPOS 100PL). The de-scanned fluorescence was focused onto a multimode fibre of 50 µm diameter and detected by an APD (PerkinElmer).

### Image processing

Maximum intensity projections were produced from raw data using the Imspector (Abberior) software. For Fig. 2c and the Supplementary Movie S1 the maximum intensity projections of the different time points were imported in Fiji^39^ to perform a stack registration (StackReg) and Bleach Correction.

### Imaging parameters

P_Exc_: Average excitation power measured in the aperture of the objective. P_STED_: Average STED power measured in the aperture of the objective. DW: Pixel dwell time. ΔX, ΔY: Pixel size in x and y, respectively.

Figure 1: (a) P_Exc_ = 2.6 µW, P_STED_ = 117 mW, DW = 25 µs, ΔX = ΔY = 30 nm

(b) P_Exc_ = 2 µW, P_STED_ = 117 mW, DW = 25 µs, ΔX = ΔY = 30 nm.

Figure 2: P_Exc_ = 13 µW, P_STED_ = 27 mW, DW = 5 µs, ΔX = ΔY = 30 nm.

Figure 3: P_Exc_ = 19 µW, P_STED_ = 50 mW, DW = 10 µs, ΔX = ΔY = 30 nm.

Figure 4: P_Exc_ = 9 µW, DW = 10 µs, ΔX = ΔY = 100 nm.

### Data availability

All data generated or analysed during this study are included in this published article (and its Supplementary Information files).

## Electronic supplementary material

Supplementary Information

Supplementary movie
