# Supplementary material for: In vivo mouse and live cell STED microscopy of neuronal actin plasticity using far-red emitting fluorescent proteins
Source: Sci Rep. 2017 Sep 18;7:11781. doi: 10.1038/s41598-017-11827-4 (PMC5603588; doi:10.1038/s41598-017-11827-4)
Supplement: Supplementary file 1 — Supplementary Information [file 41598_2017_11827_MOESM1_ESM.pdf]

Supplementary Information

In vivo mouse and live cell STED microscopy of neuronal actin plasticity using far-red emitting fluorescent proteins

Waja Wegner[1,2,3], Peter Ilgen[1,2,3], Carola Gregor[4], Joris van Dort[1,3], Alexander Mott[1,3], Heinz Steffens[1,2,3,4], Katrin I. Willig[1,2,3,*]

[1]Optical Nanoscopy in Neuroscience, Center for Nanoscale Microscopy and Molecular Physiology of the Brain, University Medical Center Göttingen, Göttingen, Germany
[2]Collaborative Research Center 889, University of Göttingen, Göttingen, Germany
[3]Max Planck Institute of Experimental Medicine, Göttingen, Germany
[4]Department of NanoBiophotonics, Max Planck Institute for Biophysical Chemistry, Göttingen, Germany

Name	Excitation max (nm)	Emission max (nm)	Quantum yield	Maturation at 37°C 50% (min)	Extinction coefficient ($M^{-1}cm^{1-}$)	Oligomerization	Ref.
Citrine	516	529	0.76		77000	Monomer	1
EGFP	488	507	0.60		56000	Monomer	2
EYFP	513	527	0.61		83400	Monomer	2
tdTomato	554	581	0.69	60	138000	tandem Dimer	3
mStrawberry	574	596	0.29	50	90000	Monomer	3
mRuby2	559	600	0.38	150	113000	Monomer	4
mRuby	558	605	0.35	170	112000	Monomer	5
FusionRed	580	608	0.19	130	94500	Monomer	6
mCherry	587	610	0.22	15	72000	Monomer	3
mRaspberry	598	625	0.15	55	86000	Monomer	7
mKate2	588	633	0.40	38	62500	Monomer	8
mNeptune2.5	599	643	0.28	26	95000	Monomer	9
mPlum	590	649	0.10	100	22000-41000	Monomer	7
mNeptune	600	650	0.23	27	67000	Monomer	9
mNeptune2	599	651	0.24	27	89000	Monomer	9
tagRFP657	611	657	0.10	125	34000	Monomer	10
mCardinal	604	659	0.19	27	87000	Monomer	9

Table S1: Evaluation of red fluorescent proteins for use in time-lapse STED microscopy.

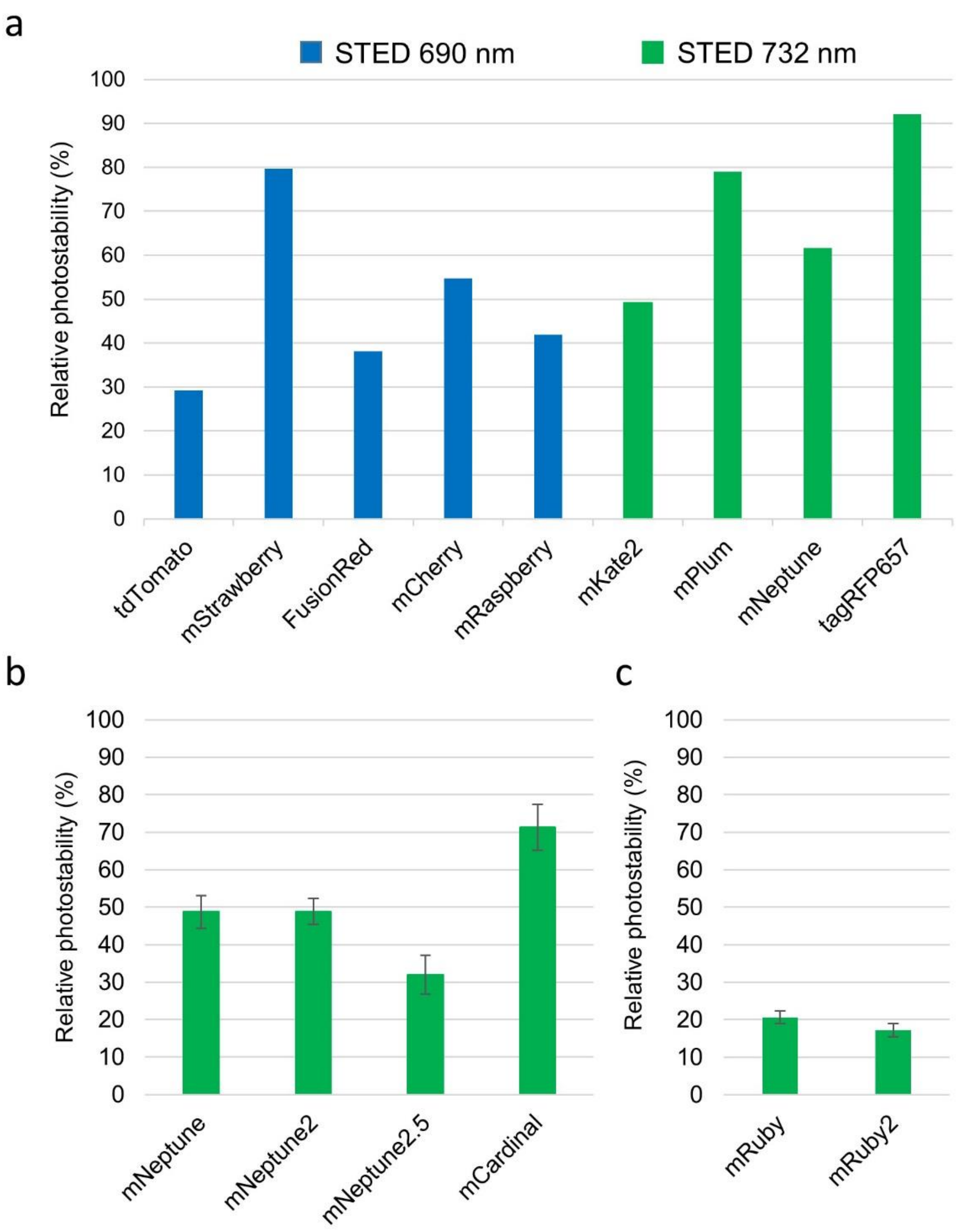

Figure S1. Photobleaching measurements by repeated STED illumination in fixed CV-1 cells. CV1-cells were transfected with different Lifeact-FP constructs and two days later fixed and imaged. Cells were scanned once with the excitation beam alone (confocal), followed by *i* consecutive scans with excitation plus STED laser. The percentage of the remaining signal after *i* rounds of scanning is shown. (**a**) For red fluorescent proteins, coloured in blue, the excitation wavelength was set to 532 nm and STED to 690 nm. Far-red proteins, illustrated in green, were investigated at 560 nm excitation wavelength, and 732 nm for STED. Excitation power was always adjusted according to their respective excitation spectrum to get equal probabilities of excitation. Relative photostability after i = 2 excitation plus STED scans (~ 20 mW) is shown as the average of n = 2 cells. (**b**) mNeptune and its derivatives were excited at 560 nm with a power of ~ 0.45 µW, followed by i = 8 consecutive scans with excitation plus STED laser (732 nm) with a power of 47.5 mW. Relative photostability is shown as the average of n = 12 cells. (**c**) Parameters for mRuby and mRuby2 were excitation at 560 nm (0.45 µW) and STED at 732 nm (9.5 mW). Relative photostability after i = 7 scans with excitation plus STED is shown as the average of n = 3 cells. Error bars represent standard deviation of the mean.

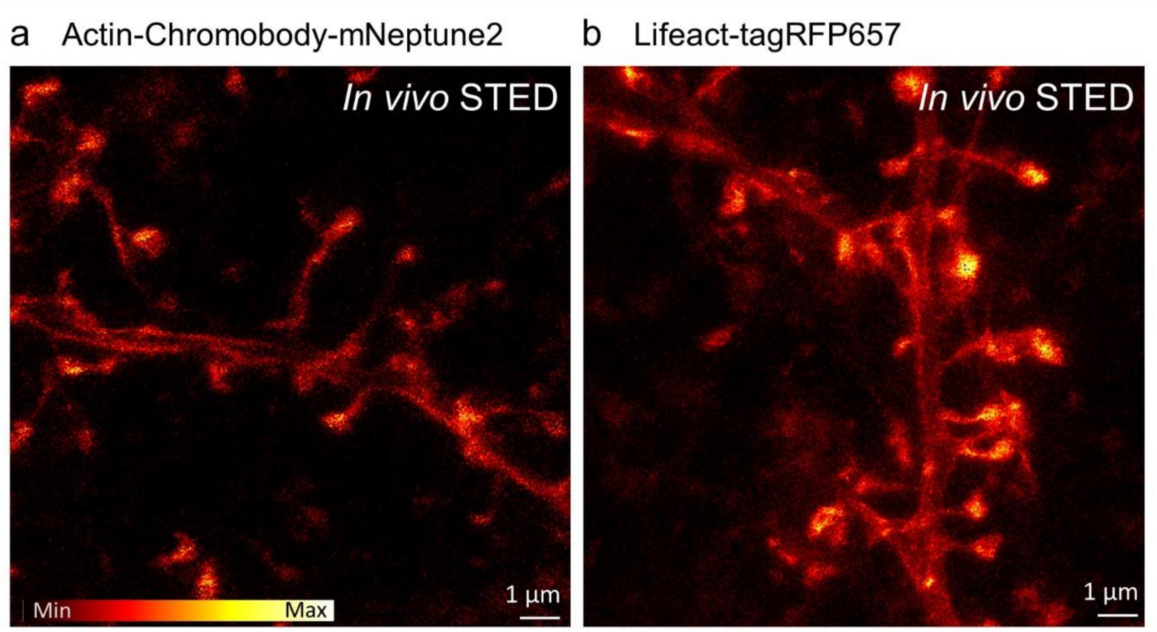

Figure S2. *In vivo* STED microscopy of filamentous actin labelled with red fluorescent proteins. (**a**) STED microscopy in L1 of the visual cortex reveals actin filaments labelled with Actin-Chromobody-mNeptune2 in dendrites and spines. (**b**) Lifeact fused to the red-emitting FP tagRFP657 reveals actin accumulation in dendritic spines in L1 of the visual cortex in the living mouse. Both images are maximum intensity projections of 5 slices of raw data.

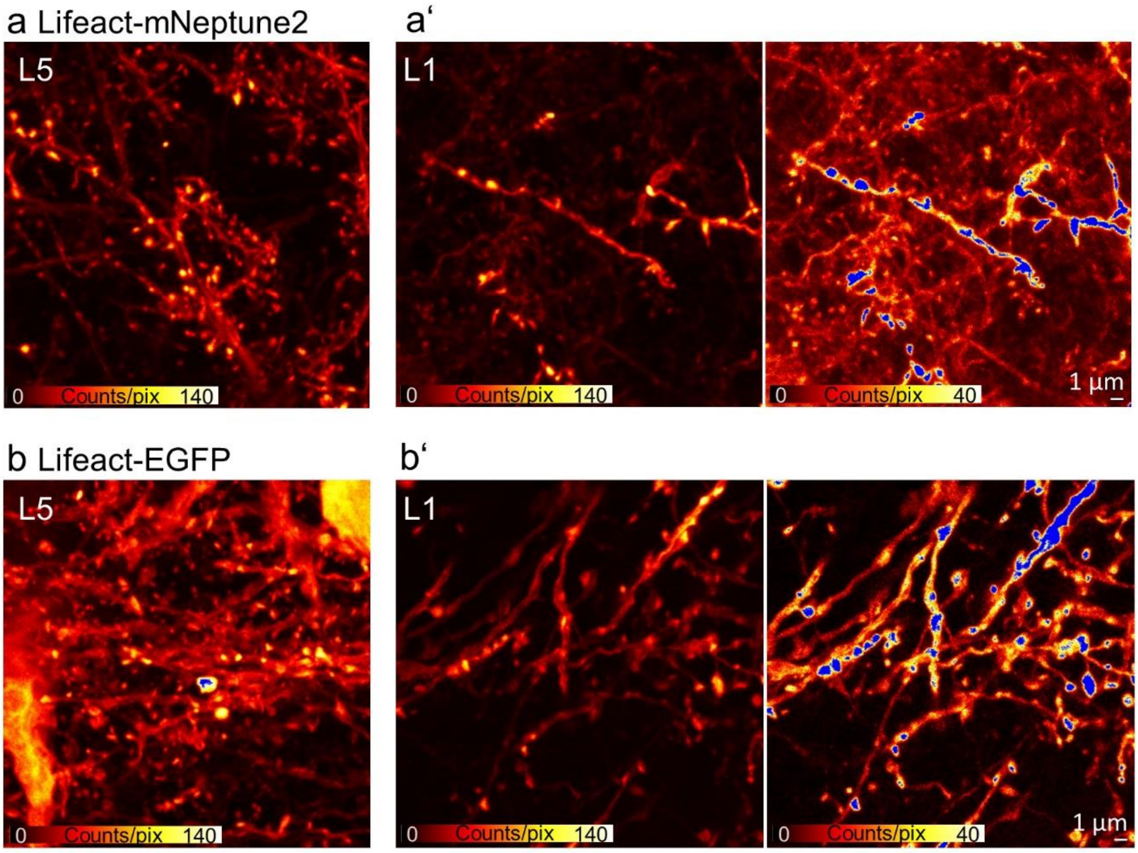

Figure S3. Fluorescence intensity of mNeptune2 actin labelling decreases at distal dendrites. Confocal images of Lifeact fused to mNeptune2 (**a**) and GFP (**b**) in brain slices of perfused mice ~ 3 weeks after AAV transduction in L5. (**a**) Dendrites are brightly fluorescent in L5. (**a’**) Only few dendrites in L1 exhibit a similar brightness as L5 dendrites; mostly the bright dendrites originate from a few bright cell bodies of L1 (not in the imaged area). Decreasing the maximum value of the colour table (**a’** right) shows a dense mesh of dendritic structures of lower brightness. (**b**) Lifeact-EGFP transduction of L5 results in bright fluorescence in L5 dendrites. (**b’**) L1 dendrites are of similar brightness as L5 dendrites. In contrast to (**a’**), changing the colour table does not reveal a dark population of dendrites (**b’** right). Blue: Saturation of the colour table. Counts/pix: Photon counts per pixel.

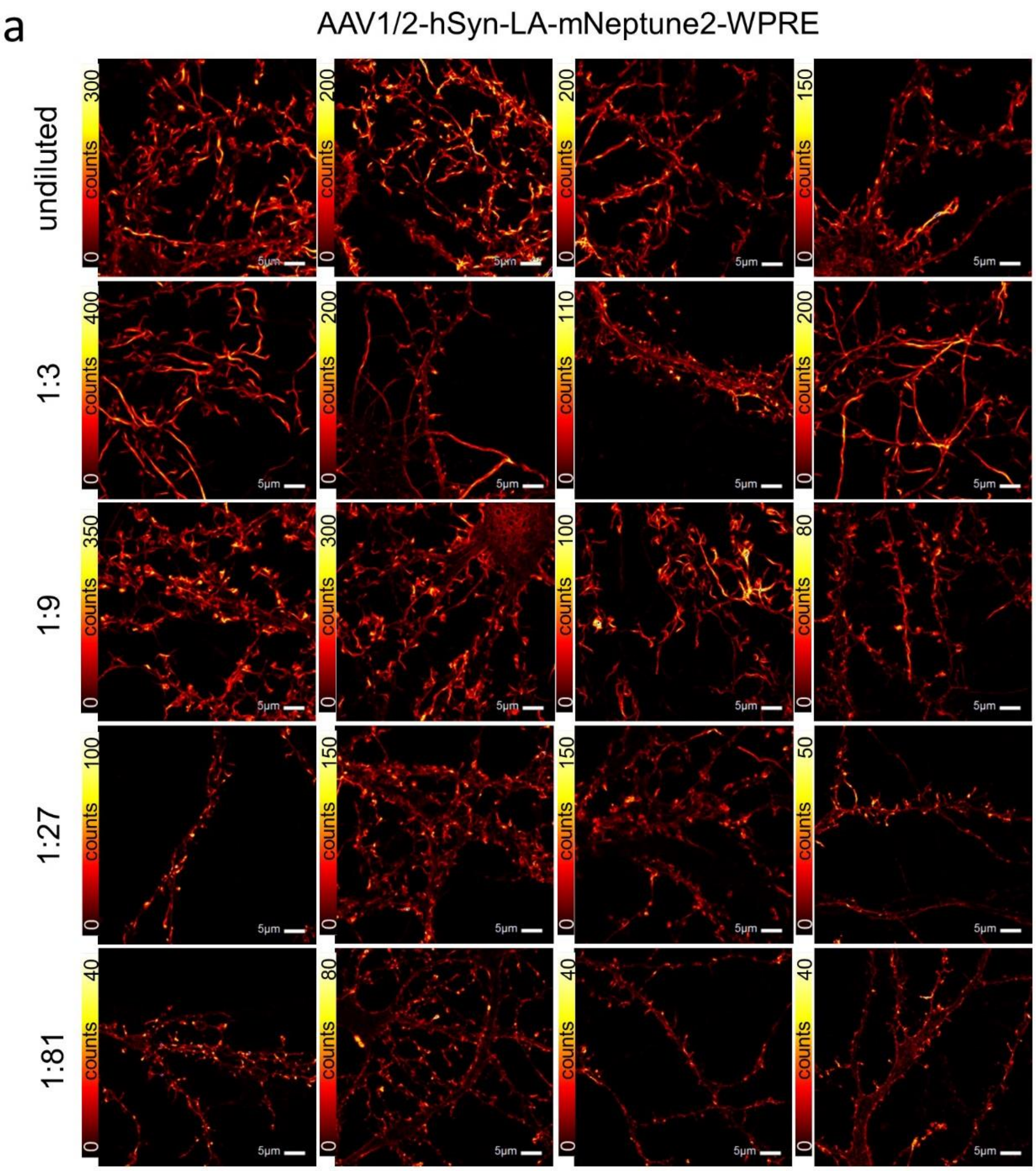
a
AAV1/2-hSyn-LA-mNeptune2-WPRE
undiluted
1:3
1:9
1:27
1:81
counts
5µm

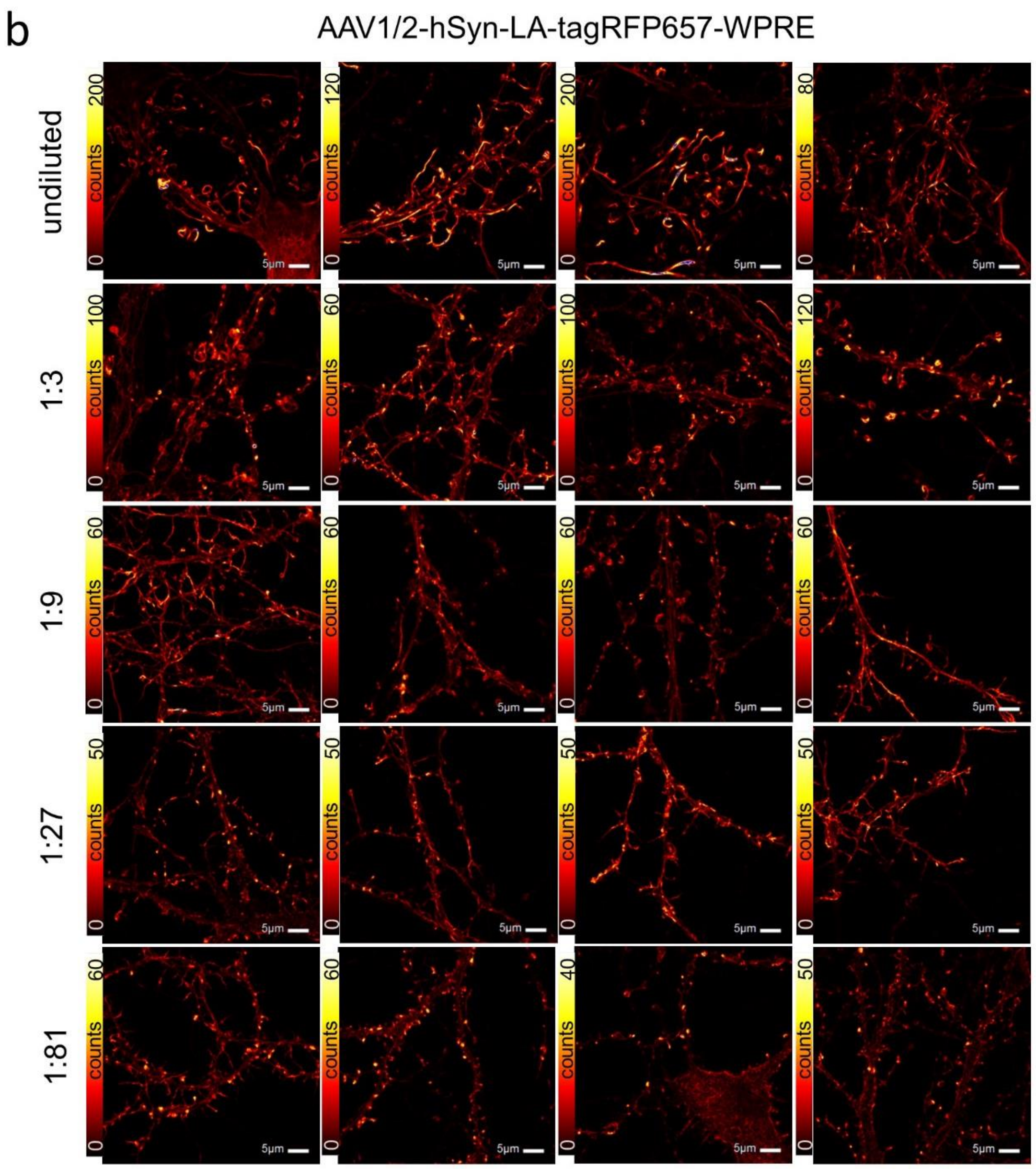
b
AAV1/2-hSyn-LA-tagRFP657-WPRE
undiluted
1:3
1:9
1:27
1:81
counts
5µm

C

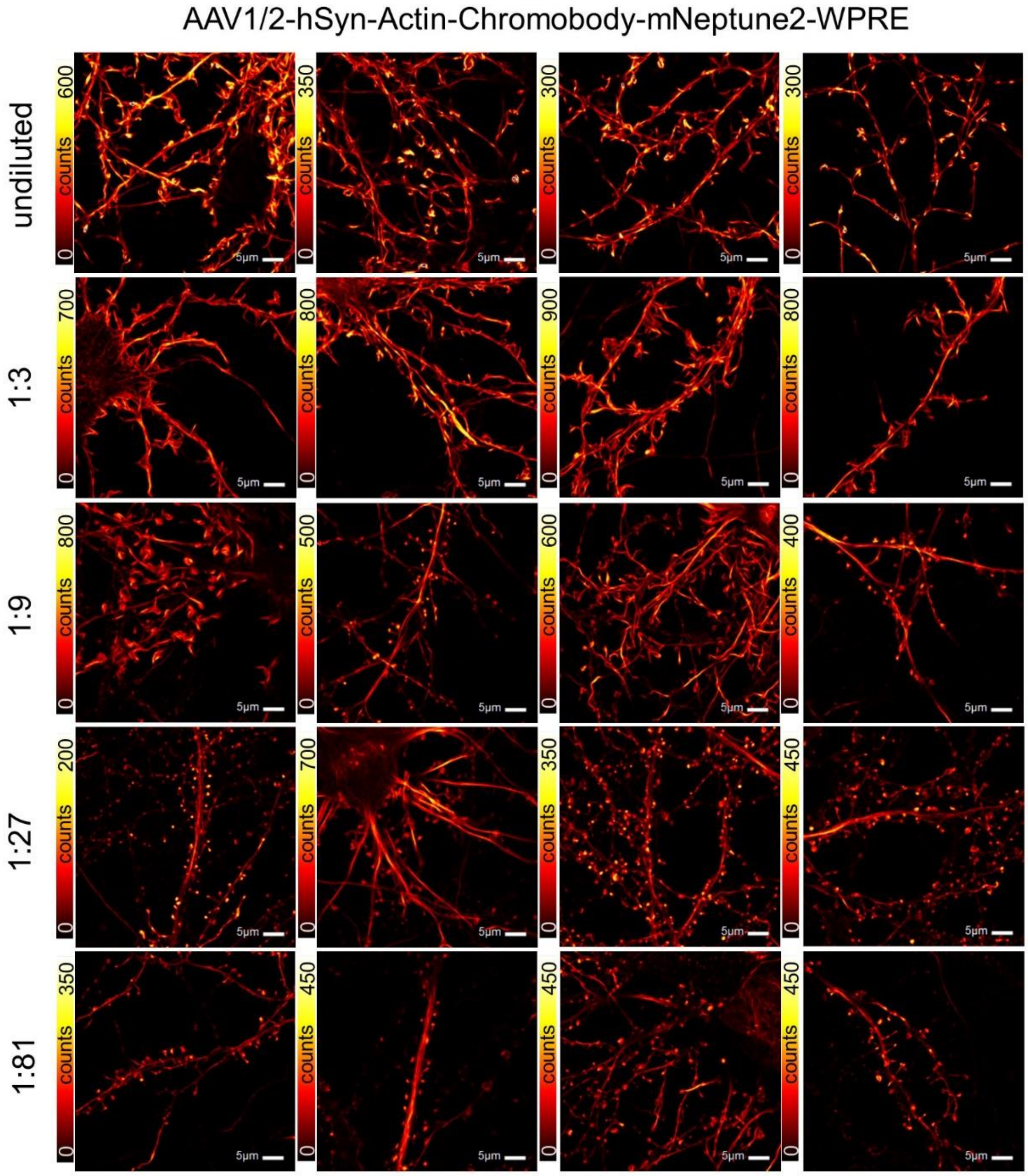
AAV1/2-hSyn-Actin-Chromobody-mNeptune2-WPRE
undiluted
1:3
1:9
1:27
1:81
counts
5µm

d

AAV1/2-hSyn-LA-EYFP-WPRE

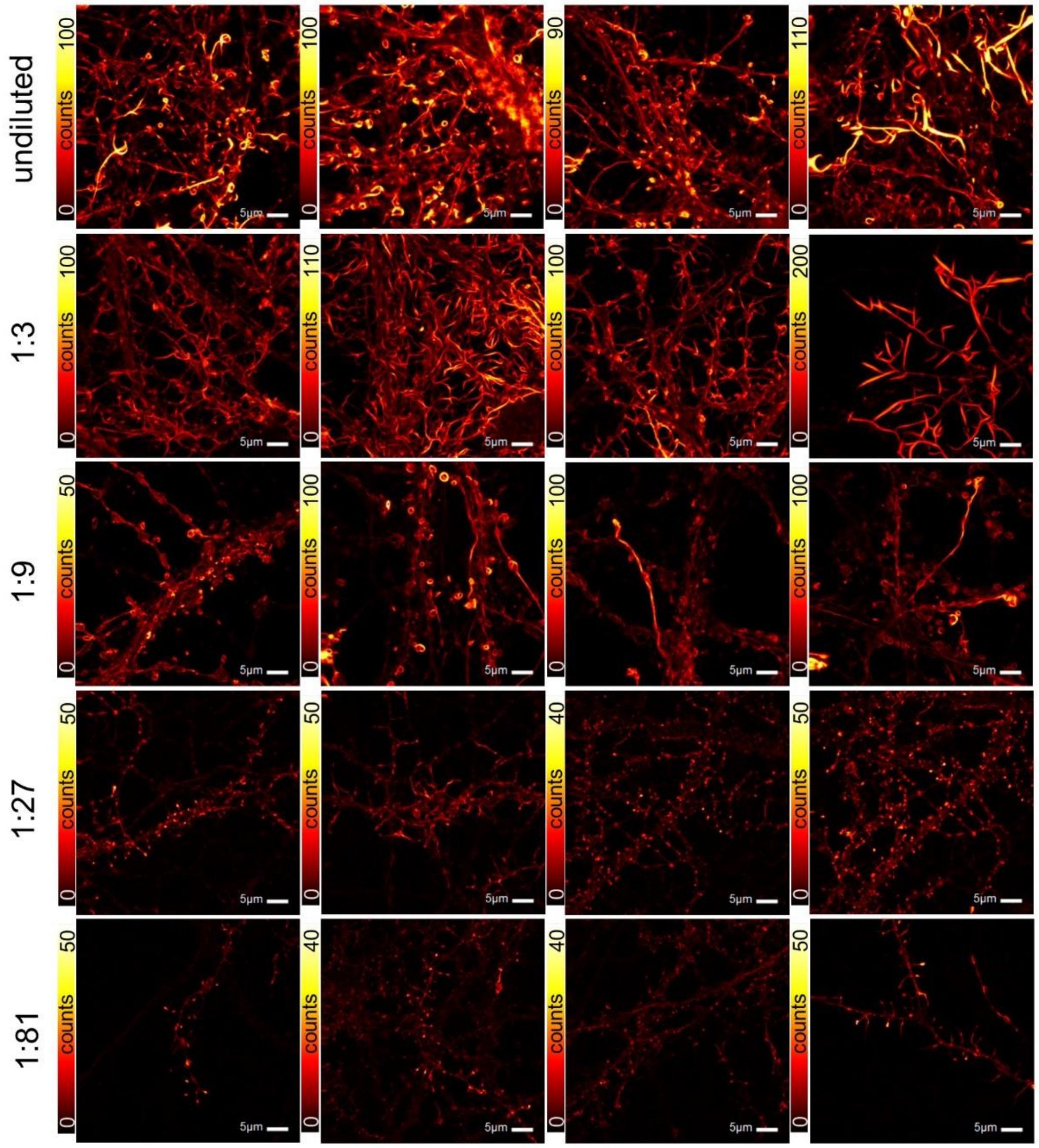

e AAV1/2-hSyn-Actin-Chromobody-EGFP-WPRE

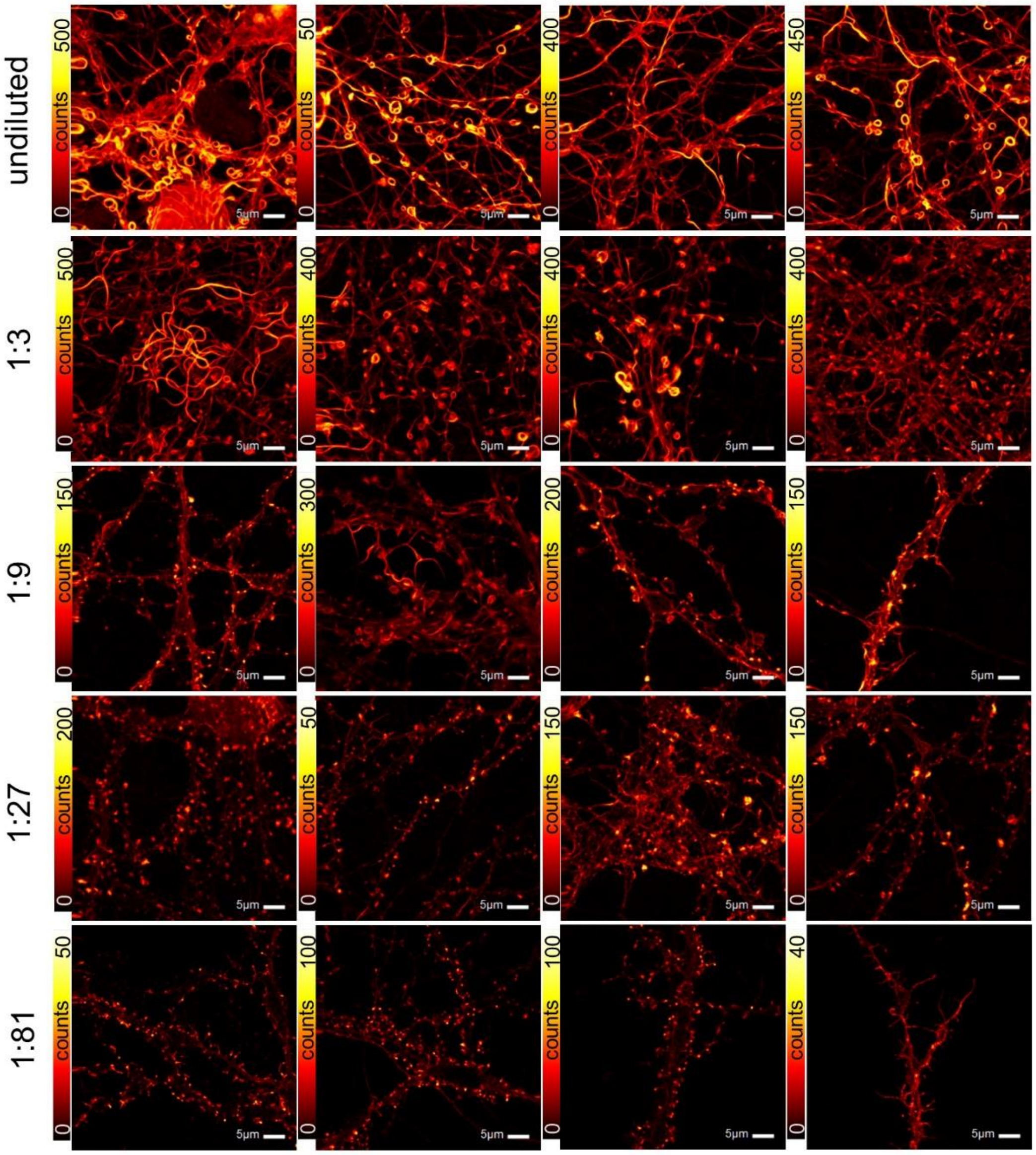

f

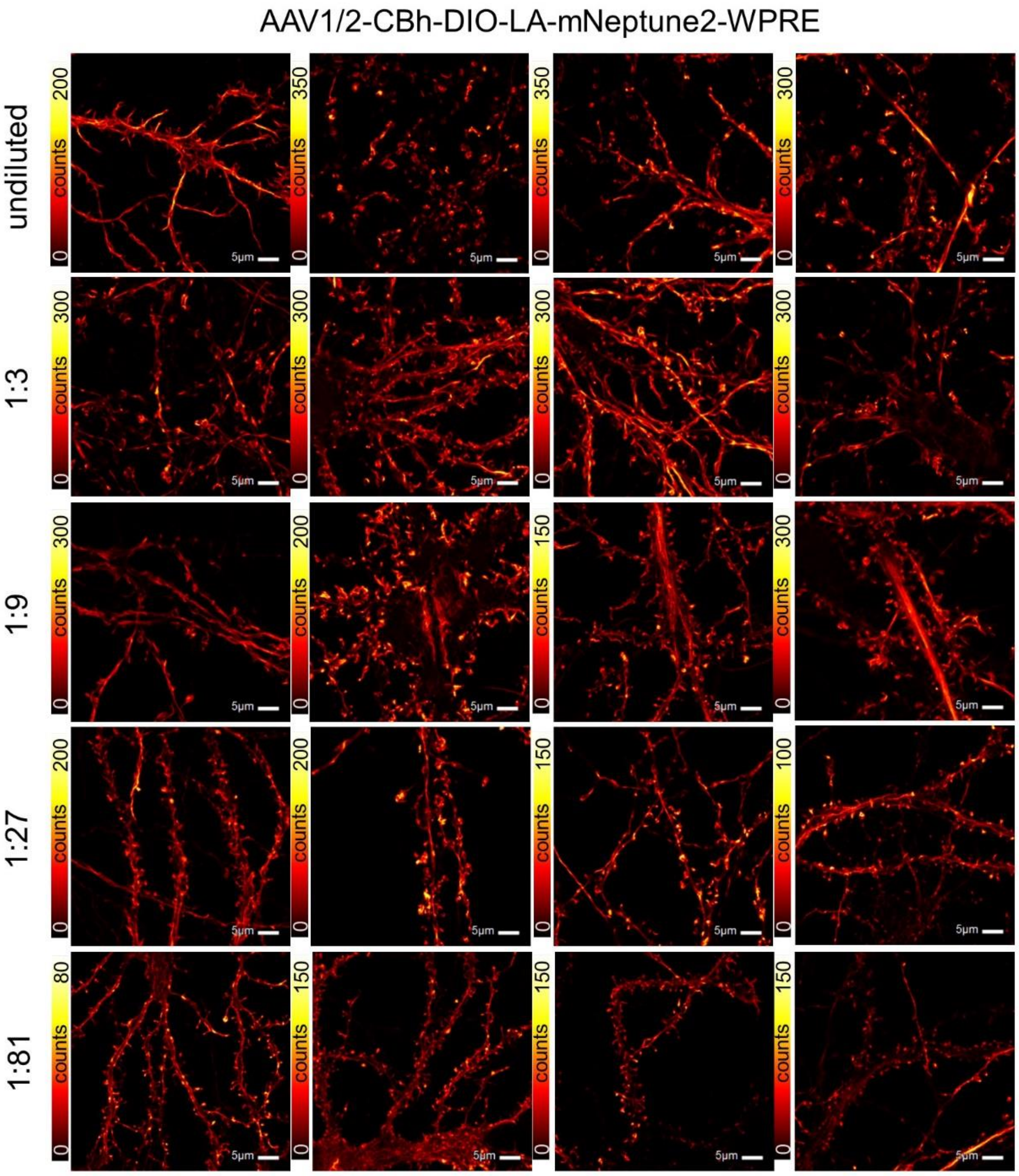
AAV1/2-CBh-DIO-LA-mNeptune2-WPRE
undiluted
1:3
1:9
1:27
1:81
counts
5µm

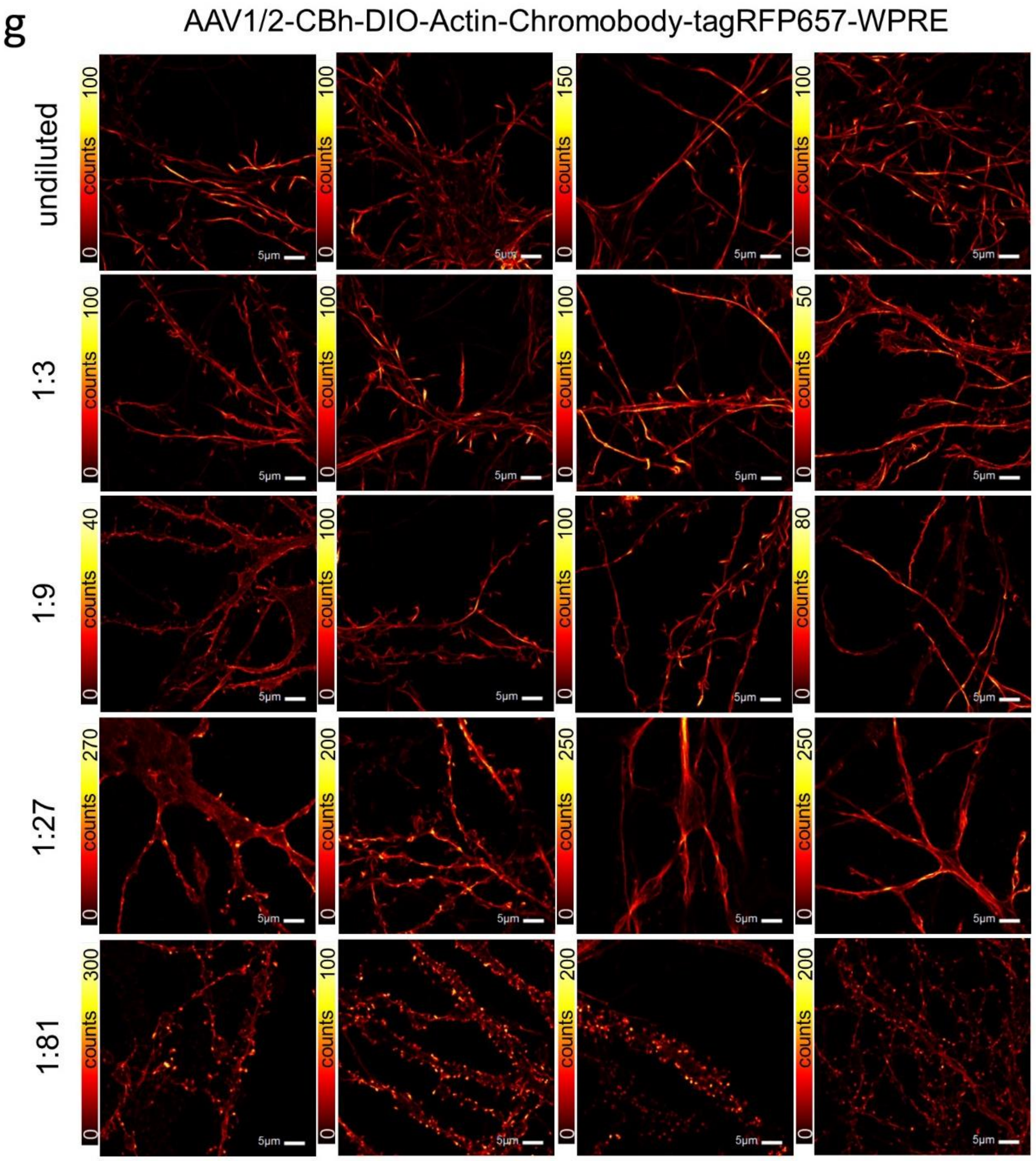

Figure S4. Spine morphology, visualized by the F-actin labels Lifeact or Actin-Chromobody fused to FPs, changes in a concentration dependent manner. Mouse or rat neurons were transduced with the indicated dilution of AAV1/2. After fixation, cells were imaged with an excitation wavelength of 586 nm (**a, b, c, f, and g**) or 490 nm (**d + e**). Reducing the concentration of AAV1/2 lead to a decrease of the overexpression induced abnormal spine elongation phenotype (**a-g**). Neurons transduced with higher dilutions showed normal spine morphology, although some neurons still have higher expression levels leading to spine elongation. (**a-e**): mouse hippocampal neurons; transduction at 8 DIV, fixation at 17 DIV; (**f + g**): rat hippocampal neurons; transduction at 17 DIV, fixation 30 DIV; (**a, b, c, f,** and **g**) data were acquired by using the live cell STED setup in confocal mode (see Methods); (**d + e**) confocal measurements as previously described[11].

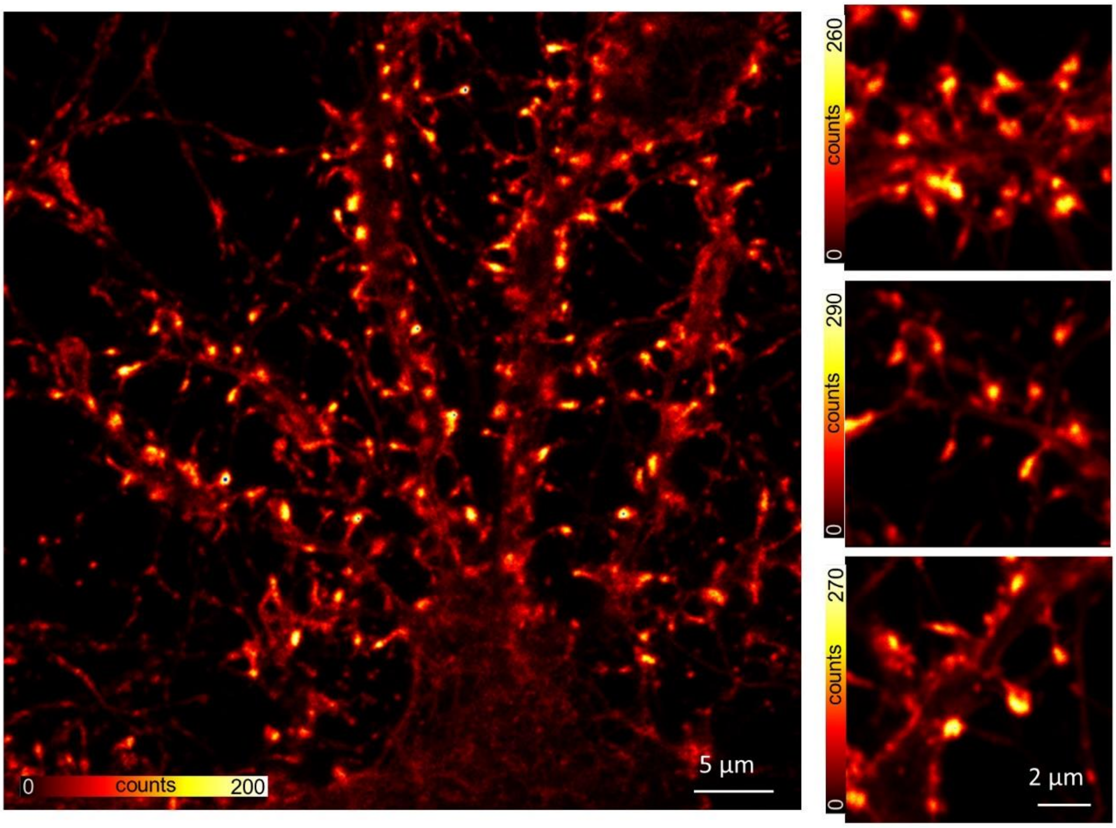

Figure S5. Phalloidin F-actin labelling in fixed hippocampal neurons. Phalloidin illustrates the actin structure in dendrites with an enrichment in spine heads, confocal image**.** Hippocampal rat neurons were fixed at 22 DIV and immediately labelled with phalloidin Atto 633. Left: overview, right: higher magnification of dendrites from neurons.

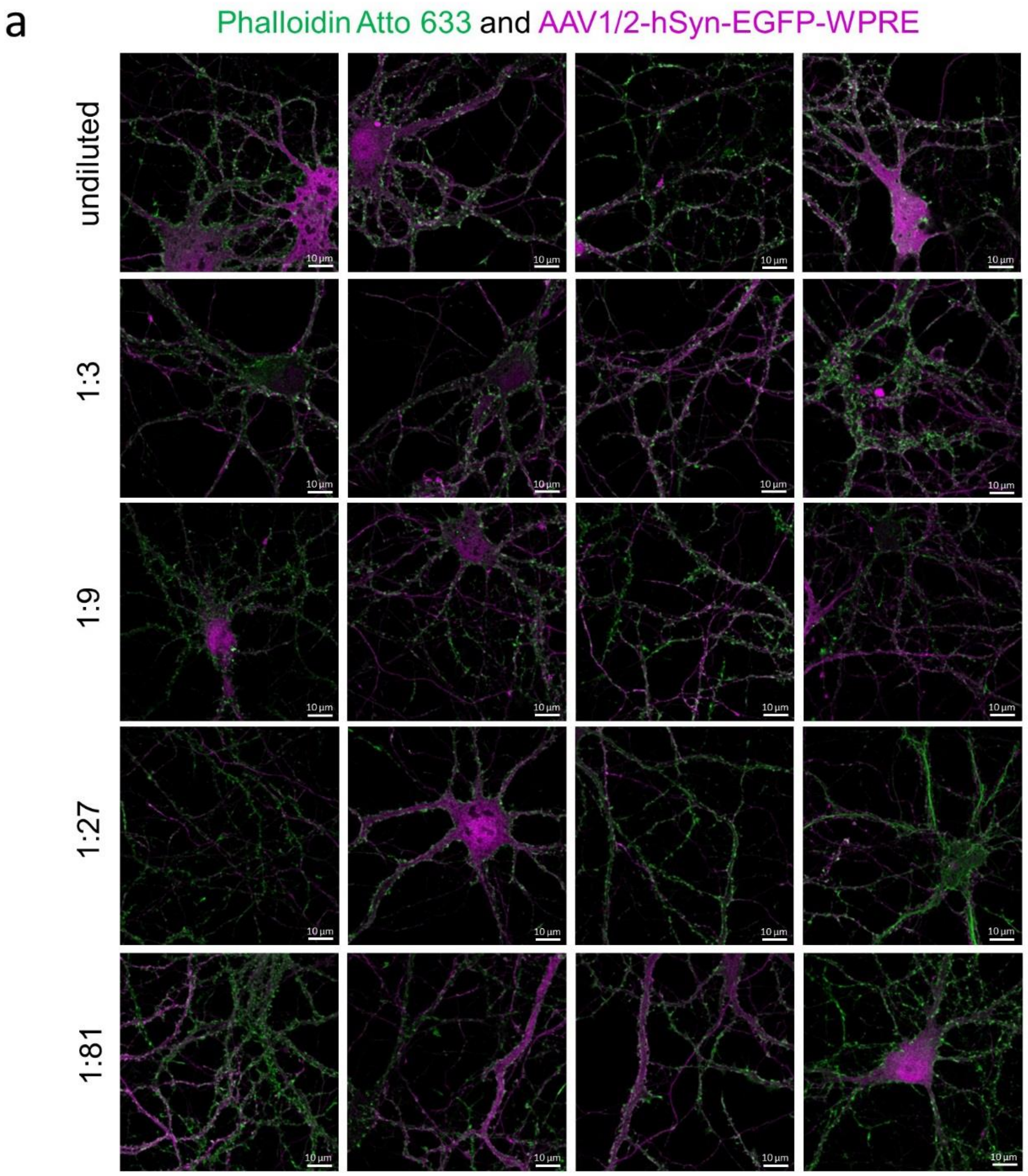
a
Phalloidin Atto 633 and AAV1/2-hSyn-EGFP-WPRE
undiluted
1:3
1:9
1:27
1:81
10 µm

b

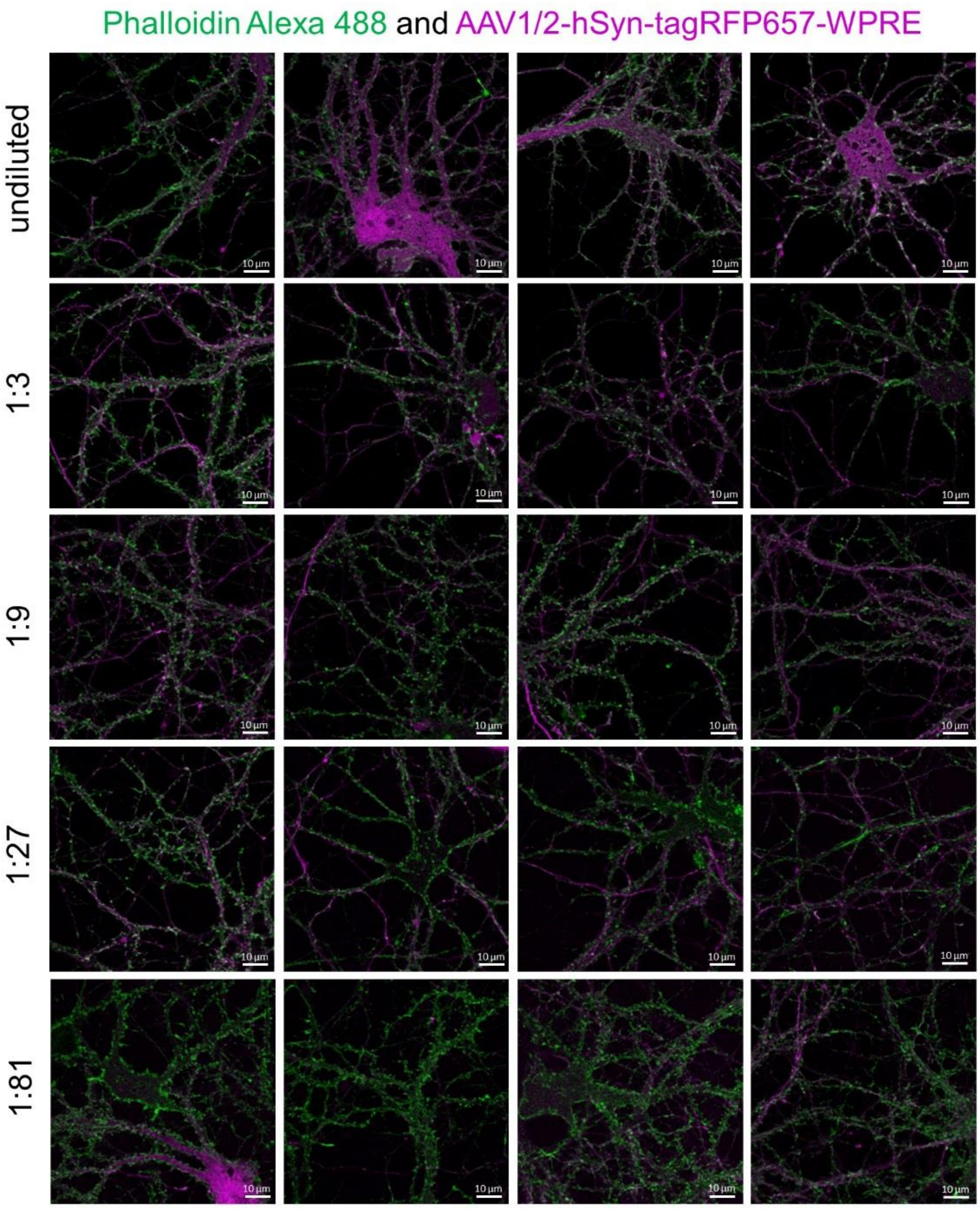
Phalloidin Alexa 488 and AAV1/2-hSyn-tagRFP657-WPRE
undiluted
1:3
1:9
1:27
1:81
10 µm

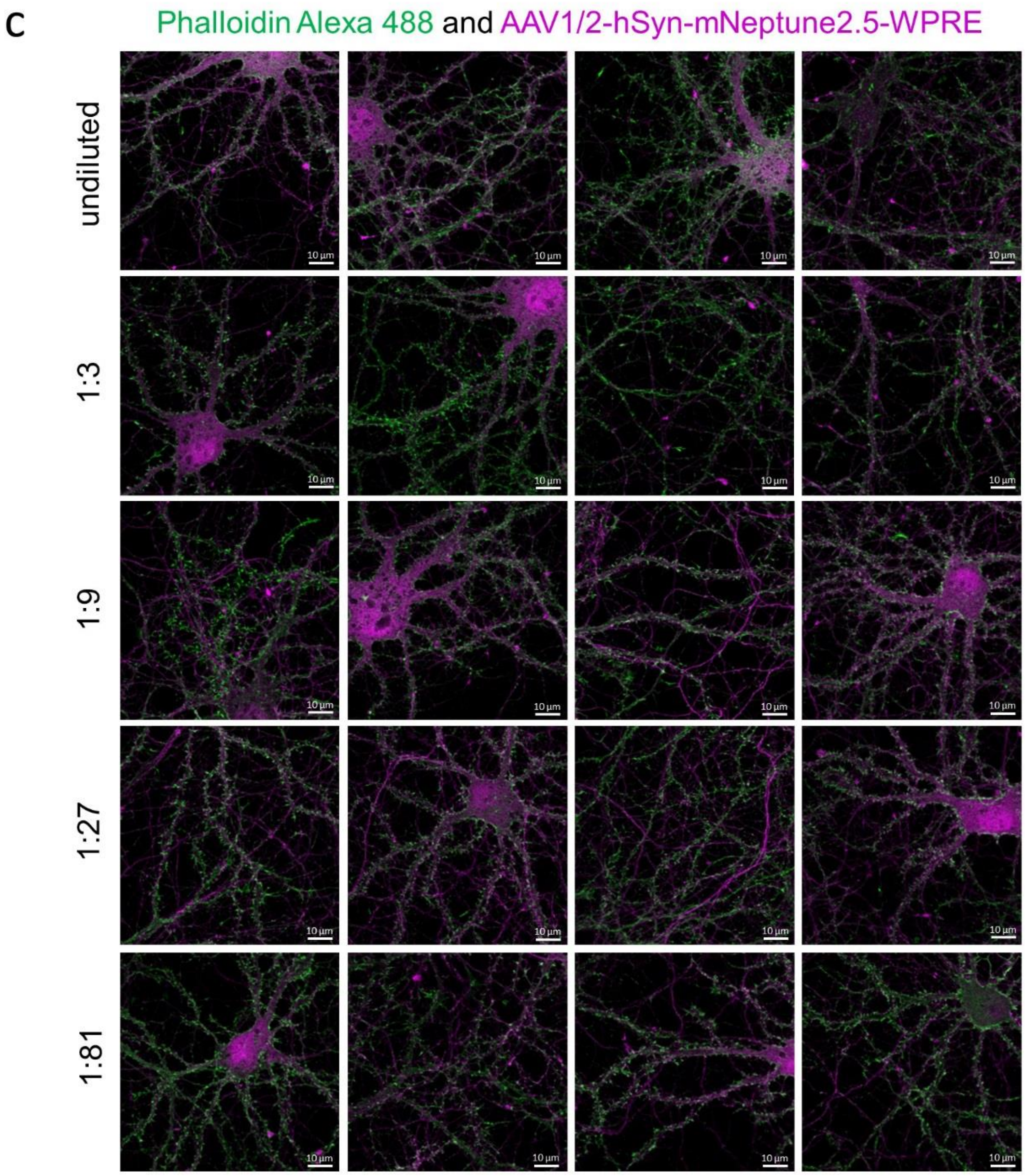

Figure S6. AAV1/2-mediated EGFP, tagRFP657, or mNeptune2.5 expression does not influence spine abundance or morphology even at high expression levels. Rat neurons were transduced with the indicated dilution of different AAV1/2s. After fixation, cells were co-labelled with either phalloidin Atto 633 (**a**) or Alexa Fluor 488 (**b, c**). All neurons were rich in spines and their abundance and morphology was comparable to non-transduced neurons shown in figure S5. AAV induced FP expression does not influence spine phenotype even at high expression levels. (**a, b**) transduction at 11 DIV, fixation at 21 DIV; (**c**) transduction at 8 DIV, fixation at 19 DIV; (**a-c**): data were acquired with the commercial Leica SP8 laser scanning confocal microscope (see Methods); scale bar: 10 µm.

Supplementary Methods

Plasmid generation

Constructs of Lifeact (LA) and different fluorescent proteins (FPs) were generated by PCR. cDNAs of all investigated FPs were amplified with 5´primers including an AgeI restriction site and 3´primers including an EcoRI restriction site, as previously described in the Methods. Purified PCR-fragments were digested and ligated into the similarly opened pAAV-hSyn-LA-EYFP plasmid creating the following constructs: pAAV-hSyn-LA-EGFP, pAAV-hSyn-LA-tdTomato, pAAV-hSyn-LA-mStrawberry, pAAV-hSyn-LA-mRuby, pAAV-hSyn-LA-mRuby2, pAAV-hSyn-LA-FusionRed, pAAV-hSyn-LA-mCherry, pAAV-hSyn-LA-mRaspberry, pAAV-hSyn-LA-mKate2, pAAV-hSyn-LA-mNeptune, pAAV-hSyn-LA-mNeptune2.5, pAAV-hSyn-LA-mCardinal, and pAAV-hSyn-LA-mPlum. Plasmids pAAV-hSyn-tagRFP657, pAAV-hSyn-mNeptune2.5 and pAAV-hSyn-EGFP encoding only the fluorescent proteins without any actin binders were generated by amplifying their corresponding cDNAs with 5´primers including a BamHI restriction site and 3´primers including an EcoRI restriction site. Digested PCR fragments were ligated into the equally digested pAAV-hSyn-LA-EYFP plasmid. To obtain the plasmid pAAV-hSyn-Chromobody-EGFP, coding sequence of EGFP was PCR amplified using forward primer 5´-GCC<u>ACTAGT</u>GGTAGTGGTGCC**ATG**GTGAGCA-3´, which adds parts of the linker region between chromobody and the fluorescent protein, as well as an internal <u>SpeI</u> restriction site to the 5´-end, and the reverse primer 5´GCTC<u>GAATTC</u>CAG**TTA**CTTGTACAGCTCG-3´ which includes an <u>EcoRI</u> restriction site. PCR-fragment was purified, digested, and ligated into the plasmid pAAV-hSyn-Chromobody-mNeptune2, which was correspondingly digested. pAAV-CBh-DIO-LA-EGFP encoding a double-floxed inverted open reading frame (DIO) of LA-EGFP and including the CBh promoter, was cloned by amplifying LA-EGFP using the forward primer 5´-GGC<u>GCTAGC</u>**ATG**GGTGTCGCAGATTTGATCAAG-3´ including a <u>NheI</u> restriction site and the reverse primer 5´-TGAATGGAATTC<u>GGCGCGCC</u>**TTA**CTTGTACAGCTCGTCC-3´ including an <u>AscI</u> restriction site. The digested PCR product was ligated into pAAV-Ef1a-DIO-EYFP-WPRE-pA provided by the laboratory of Dr. K. Deisseroth of Stanford University, CA. digested with NheI and AscI, resulting in pAAV-Ef1a-DIO-LA-EGFP. To exchange the hSyn promoter to CBh, CBh was amplified from the plasmid pX330-U6-Chimeric_BB-CBh-hSpCas9 (a gift from Dr. Feng Zhang, Addgene plasmid #42230[12]), by PCR using the forward primer 5'-TGATTA<u>ACGCGT</u>CGTTACATAACTTACGGTAAATGGC-3' and the reverse primer 5'-TTACTA<u>GGTACC</u>CCAACCTGAAAAAAAGTGATTTCAGG-3', and cloned into pAAV-Ef1a-DIO-LA-EGFP with <u>MluI</u> and <u>KpnI</u>, resulting in pAAV-CBh-DIO-LA-EGFP. The plasmid pAAV-CBh-DIO-LA-mNeptune2 was cloned by using forward primer 5´-GGC<u>GCTAGC</u>**ATG**GGTGTCGCAGATTTGATCAAG-3´ including a <u>NheI</u> restriction site, and reverse primer 5´-CAT<u>GGCGCGCC</u>**TTA**CTTGTACAGCTCGTCCATG-3´ adding an <u>AscI</u> site, to amplify LA-mNeptune2. The purified and digested PCR product was ligated into the equally opened pAAV-CBh-DIO-LA-EGFP construct. For the generation of pAAV-CBh-DIO-Actin-Chromobody-tagRFP657, two PCRs were performed. In the first PCR Actin-Chromobody, including the linker sequence, was amplified with the forward primer 5'-GCAT<u>GCTAGC</u>**ATG**GCTCAGGTGCAGCTGGT-3' (<u>NheI</u>) and the reverse primer 5´- TCCTTAATCAGCTCTTCGCCCTTAGACAC-3´, using pQE30-Chromobody-mNeptune2 (see Methods) as a template. In a second PCR, cDNA of tagRFP657 was amplified by using forward primer 5´-*AGCC<u>ACTAGT</u>GGTAGTGGTGCC***ATG**AGCGAGCTGATCAC-3´ adding parts of the linker (*italic style*), and <u>*SpeI*</u> restriction site, as well as the reverse primer 5´-TAT<u>GGCGCGCC</u>**TTA**ATTAAGCTTGTGCCCCAG-3´ (<u>AscI</u>). After purification and digestion with NheI, SpeI, and AscI, PCR fragments were ligated into the NheI and AscI digested pAAV-CBh-DIO-LA-EGFP vector. The Cre recombinase expression plasmid pAAV-hSyn-Cre, was cloned by amplifying the coding sequence of Cre recombinase out of the plasmid pAAV-Ef1a-mCherry-IRES-WGA-Cre (kindly provided

by the laboratory of Dr. K. Deisseroth), using the forward primer 5´-ATTAGGATCC**ATG**CCCAAGAAGAAGAGGAAGGTGT-3´ (BamHI) and the reverse primer 3´-ATTGAATTC**TCA**GTCACCATCTTCGAGCAGTCTCA-5 (EcoRI). After purification and digestion, PCR fragment was cloned into the equally digested plasmid pAAV-hSyn-LA-EYFP.

Neuronal cell culture, transduction, fixation, labelling, and imaging

Transduction of neurons with different AAVs at 8-20 DIV was carried out by adding 1 µl of the virus or the indicated dilutions (1:3, 1:9, 1:27, and 1:81 in PBS pH 7.4) directly onto the neurons in a 12-well. The amount of Cre-expressing virus was always 1 µl. Fixation and labelling was performed at an age of 17-30 DIV. Fixation procedure was the following: cells were washed with PBS (pH 7.4), fixed for 20 min in 4 % PFA (in PBS pH 7.4) at room temperature, washed three times with PBS, and finally embedded in Mowiol 4-88 mounting medium (Carl Roth GmbH, cat. no. 0713, Karlsruhe, Germany). Phalloidin labelling was performed as follows: 4 % PFA-fixation was quenched for 5 min with ammonium chloride and glycine (100 mM each), followed by 5 min permeabilization using 0.1 % Triton X-100 in PBS (pH 7.4). Samples were further blocked with 1 % BSA in PBS (pH 7.4) for 30 min and labelled for 1 h with either phalloidin Atto 633 (ATTO-TEC GmbH, Siegen, Germany) or phalloidin Alexa Fluor 488 (Molecular Probes, Thermo Fisher Scientific, Darmstadt, Germany) in PBS (pH 7.4). Finally, samples were rinsed three times with PBS (pH 7.4) and mounted with Mowiol 4-88.

CV-1 cell culture, transfection, fixation, and imaging

Adherent CV-1 cells (ATCC® CCL-70™, Manassas, VA) were cultured in DMEM high glucose GlutaMAX™ (Gibco, cat. no. 61965026, Thermo Fisher Scientific) supplemented with 1 % sodium pyruvate (Sigma-Aldrich cat. No. S8636, Darmstadt, Germany), 10 % FBS (Biochrom GmbH cat. no. S 0615, Berlin, Germany), and penicillin/streptomycin (100 units per milliliter and 100 µg/mL respectively) (Biochrom GmbH, cat. no. A 2213) at 37 °C and 5 % CO_2 humidified atmosphere. CV-1 cells were transfected with pAAV-hSyn-LA-FP at a density of ~ 80 % by using TurboFect™ (Thermo Fisher Scientific) according to manufacturer's recommendations. Two days after transfection, cells were washed twice with PBS (pH 7.4), fixed for 15 min in 4 % PFA in PBS (pH 7.4), rinsed three times with PBS (pH7.4), and embedded in Mowiol 4-88 mounting medium. To determine relative photostability, fluorescence intensity (F_i) over time was investigated. Hence CV-1 cells were illuminated as follows: one time excitation beam alone (confocal before STED), followed by several continuous illuminations of excitation plus STED beam (STED mode), closing with one time excitation beam alone (confocal after STED). Relative photostability was calculated in percent as follows: relative photostability [%] = (F_i (confocal after STED) * 100) / F_i (confocal before STED)).

Two-colour confocal microscopy

Two-colour confocal microscopy of fluorescent proteins with the corresponding phalloidin was performed using a commercial Leica SP8 laser scanning confocal microscope (Leica, Wetzlar, Germany), equipped with a 63 x oil objective (Leica). Images were acquired by using an excitation wavelength of 488 nm for EGFP and phalloidin Alexa Fluor 488, and 633 nm for phalloidin Atto 633, tagRFP657, and mNeptune2.5. Fluorescence was detected with a PMT in a range of 495-600 nm for 488 nm excitation and 640-800 nm for 633 nm excitation. Imaging was done with a line average of 3 and a scan field of 1304 x 1304 pixels. Images were smoothed with Imspector software (Abberior instruments) by using smoothing type lowpass (gauss).

Imaging parameters

P_{Exc}: Average excitation power measured in the aperture of the objective. P_{STED}: Average STED power measured in the aperture of the objective. DW: Pixel dwell time. ΔX, ΔY: Pixel size in x and y, respectively.

Figure S2: a) P_{Exc} = 18 µW, P_{STED} = 33 mW, DW = 6 µs, ΔX = ΔY = 30 nm
Figure S2: b) P_{Exc} = 14 µW, P_{STED} = 21 mW DW = 20 µs, ΔX = ΔY = 30 nm
Figure S3: a) P_{Exc} = 18 µW, DW = 5 µs, ΔX = ΔY = 30 nm
Figure S3: b) P_{Exc} = 3.7 µW, DW = 5 µs, ΔX = ΔY = 30 nm
Figure S4: a) P_{Exc} = 0.4 µW, DW = 50 µs, ΔX = ΔY = 100 nm
Figure S4: b) P_{Exc} = 0.4 µW, DW = 50 µs, ΔX = ΔY = 100 nm
Figure S4: c) P_{Exc} = 0.4 µW, DW = 50 µs, ΔX = ΔY = 100 nm
Figure S4: d) P_{Exc} = 0.5 µW, DW = 10 µs, ΔX = ΔY = 100 nm
Figure S4: e) P_{Exc} = 1.9 µW, DW = 10 µs, ΔX = ΔY = 100 nm
Figure S4: f) P_{Exc} = 0.2 µW, DW = 50 µs, ΔX = ΔY = 100 nm
Figure S4: g) P_{Exc} = 0.2 µW, DW = 50 µs, ΔX = ΔY = 100 nm (undiluted - 1:9)
P_{Exc} = 1.8 µW, DW = 50 µs, ΔX = ΔY = 100 nm (1:27 - 1:81)
Figure S5: P_{Exc} = 0.2 µW, DW = 50 µs, ΔX = ΔY = 100 nm (overview) or 20 nm (magnification)

Supplementary references

1. Griesbeck, O., Baird, G. S., Campbell, R. E., Zacharias, D. a. & Tsien, R. Y. Reducing the environmental sensitivity of yellow fluorescent protein. Mechanism and applications. *J. Biol. Chem.* **276,** 29188–29194 (2001).

2. Tsien, R. Y. the Green Fluorescent Protein. *Annu. Rev. Biochem.* **67,** 509–544 (1998).

3. Shaner, N. C. *et al.* Improved monomeric red, orange and yellow fluorescent proteins derived from Discosoma sp. red fluorescent protein. *Nat. Biotechnol.* **22,** 1567–1572 (2004).

4. Lam, A. J. *et al.* Improving FRET dynamic range with bright green and red fluorescent proteins. *Nat. Methods* **9,** 1005–1012 (2012).

5. Kredel, S. *et al.* mRuby, a bright monomeric red fluorescent protein for labeling of subcellular structures. *PLoS One* **4,** 1–7 (2009).

6. Shemiakina, I. I. *et al.* A monomeric red fluorescent protein with low cytotoxicity. *Nat. Commun.* **3,** 1204 (2012).

7. Wang, L., Jackson, W. C., Steinbach, P. a. & Tsien, R. Y. Evolution of new nonantibody proteins via iterative somatic hypermutation. *Proc. Natl. Acad. Sci.* **101,** 16745–16749 (2004).

8. Shcherbo, D. *et al.* Far-red fluorescent tags for protein imaging in living tissues. *Biochem. J.* **418,** 567–574 (2009).

9. Chu, J. *et al.* Non-invasive intravital imaging of cellular differentiation with a bright red-excitable fluorescent protein. *Nat. Methods* **11,** 572–578 (2014).

10. Morozova, K. S. *et al.* Far-red fluorescent protein excitable with red lasers for flow cytometry and superresolution STED nanoscopy. *Biophys. J.* **99,** L13–L15 (2010).

11. Willig, K. I. *et al.* Nanoscopy of Filamentous Actin in Cortical Dendrites of a Living Mouse. *Biophys. J.* **106,** L01–L03 (2014).

12. Cong, L. *et al.* Multiplex Genome Engineering Using CRISPR/VCas Systems. *Science* **339,** 819–823 (2013).
